# Supplementary material for: Encapsulation mechanisms and structural studies of GRM2 bacterial microcompartment particles
Source: Nat Commun. 2020 Jan 20;11:388. doi: 10.1038/s41467-019-14205-y (PMC6971018; doi:10.1038/s41467-019-14205-y)
Supplement: Supplementary file 1 — Supplementary information [file 41467_2019_14205_MOESM1_ESM.pdf]

Encapsulation mechanisms and structural studies of GRM2 bacterial microcompartment particles

Kalnins et. al.

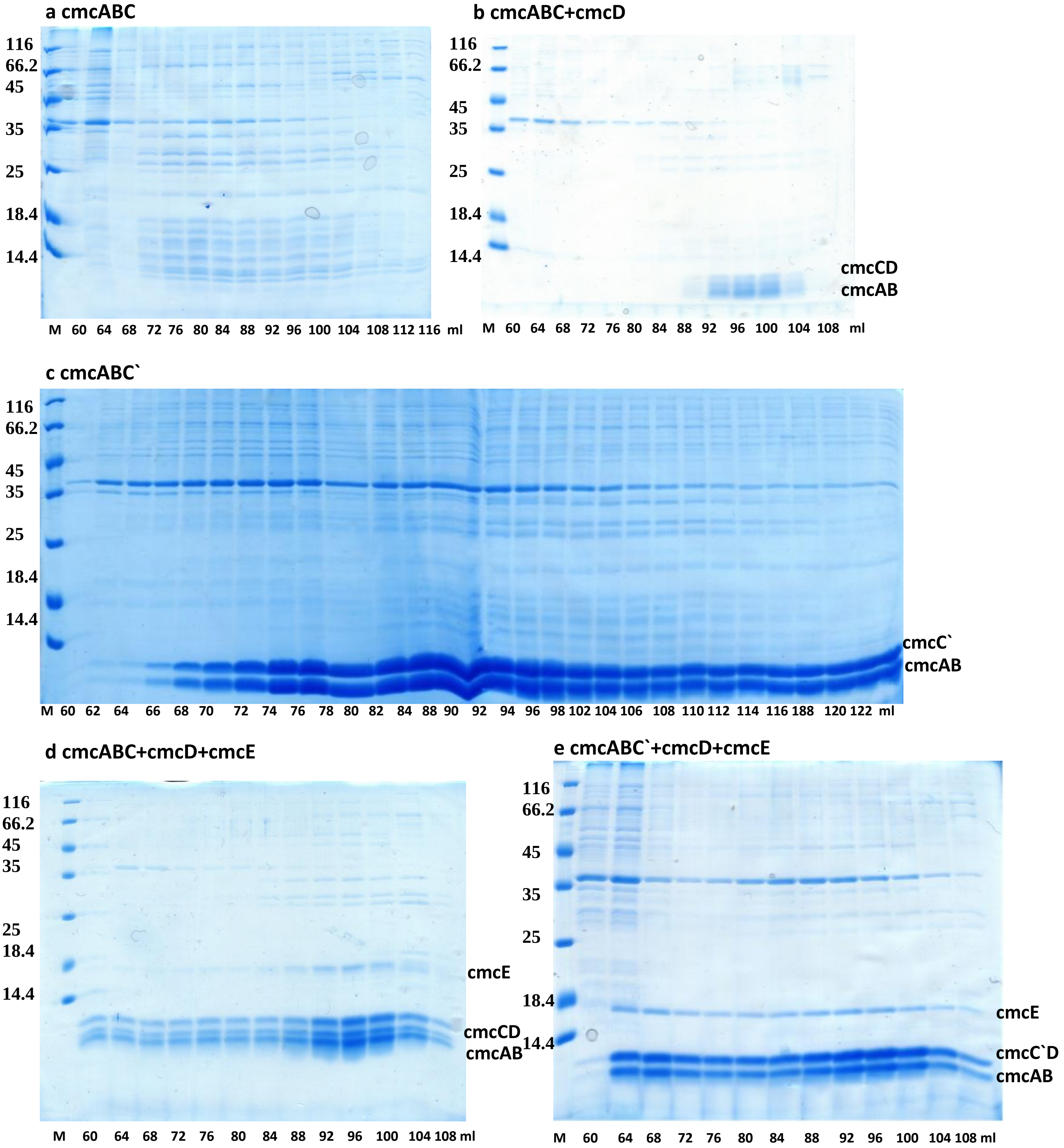

Supplementary Figure 1 SDS-PAGE analysis of Superose 6 gel filtration chromatography processes. a, cmcABC 60-112 ml. **b**, cmcABC+cmcD 60-116 ml. **c,** cmcABC`60-122 ml, corresponding protein bands are identified. **d,** cmcABC+cmcD+cmcE 60-108 ml. **e,** cmcABC`+cmcD+cmcE 60-108 ml, corresponding protein bands are identified. M – marker, sizes are measured in kDa.

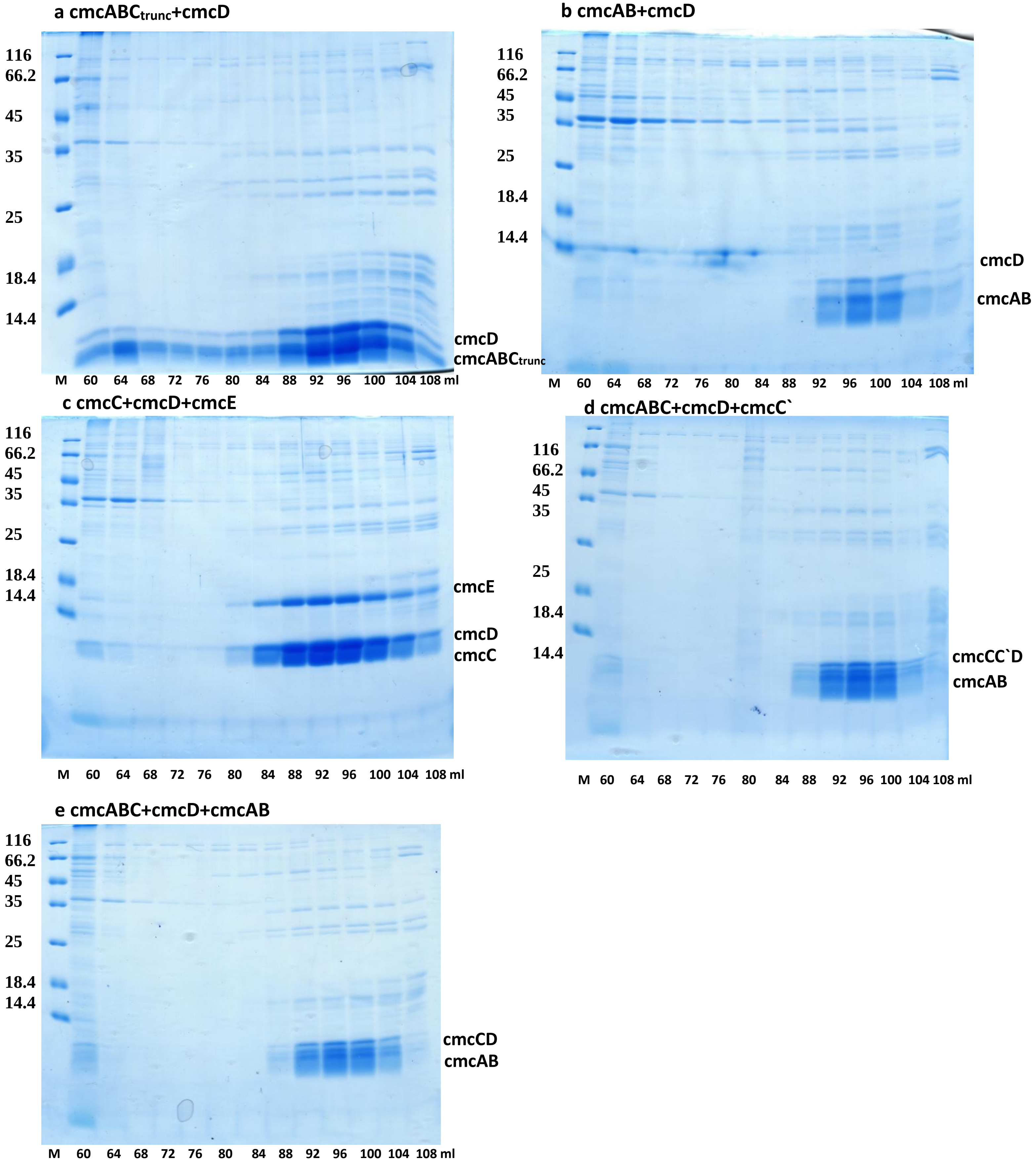

Supplementary Figure 2 SDS-PAGE analysis of Superose 6 gel filtration chromatography processes. a, cmcABC$_{trunc}$+D 60-108 ml. **b,** cmcAB+cmcD 60-108 ml. **c,** cmcC+cmcD+cmcE 60-108 ml. **d,** cmcABC+cmcD+cmcC` 60-108 ml. **e,** cmcABC+cmcD+cmcAB 60-108 ml. M – marker, sizes are measured in kDa.

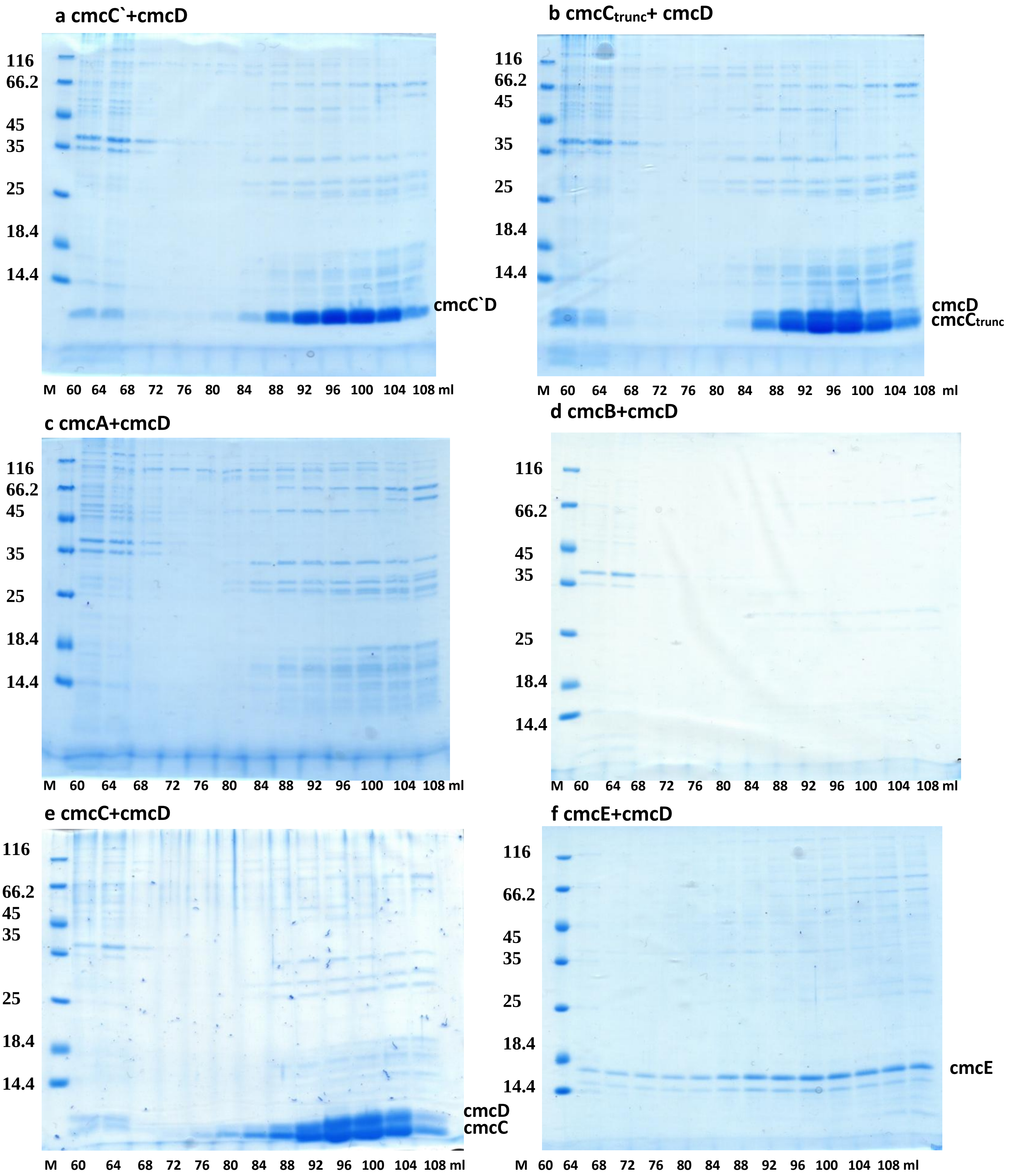

Supplementary Figure 3 SDS-PAGE analysis of Superose 6 gel filtration chromatography processes. a, cmcC`+cmcD 60-108 ml. **b**, cmcC$_{trunc}$+ cmcD 60-108 ml. **c**, cmcA+ cmcD 60-108 ml. **d,** cmcB+ cmcD 60-108 ml. **e,** cmcC+ cmcD 60-108 ml. **f,** cmcE+ cmcD 60-108 ml. M – marker, sizes are measured in kDa.

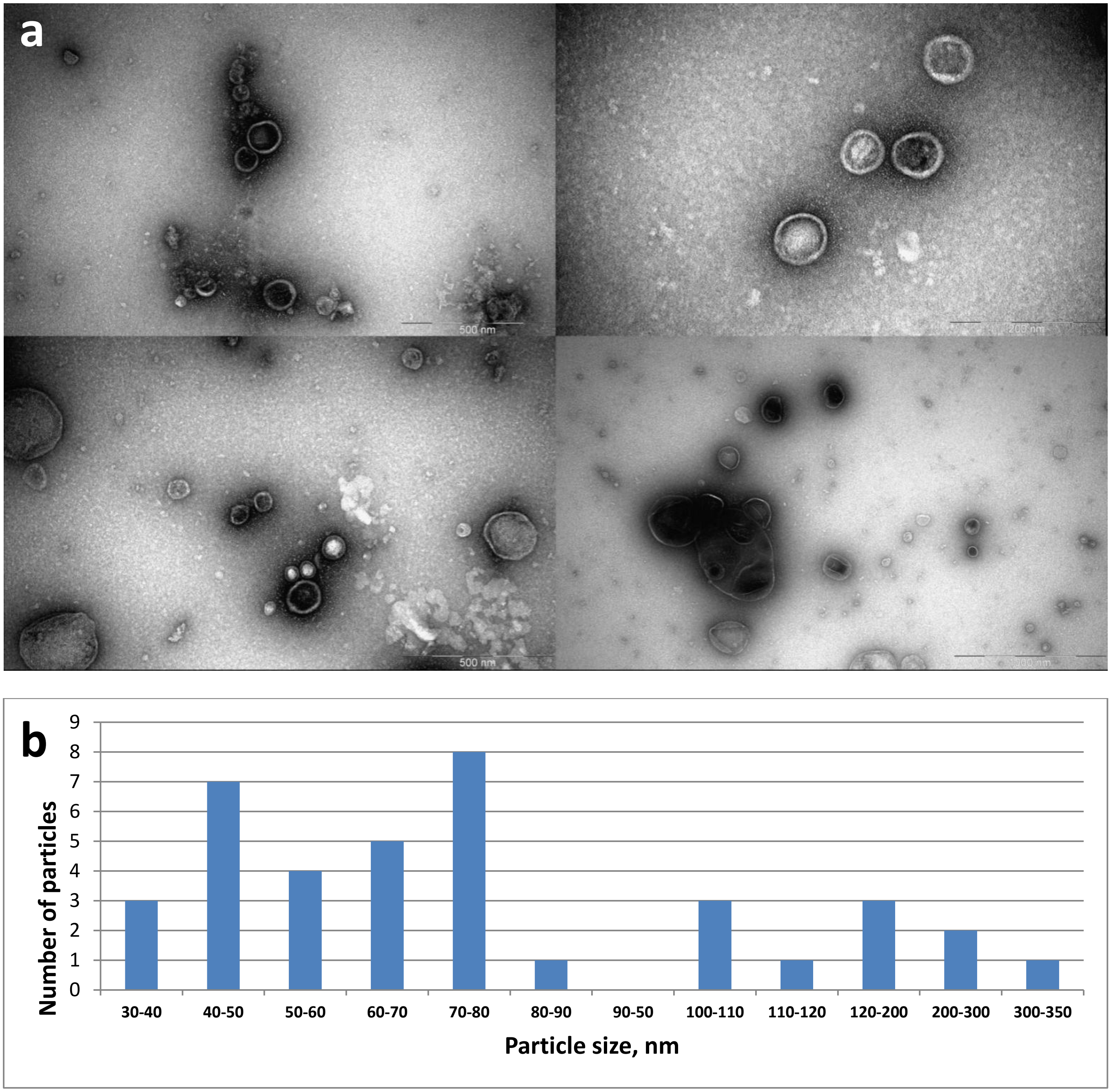

Supplementary Figure 4 TEM analysis of Superose 6 gel filtration 60-62 ml fraction of cmcABC`+D BDPs. a, negative staining TEM micrographs. **b,** particle size distribution, determined with ImageJ software.

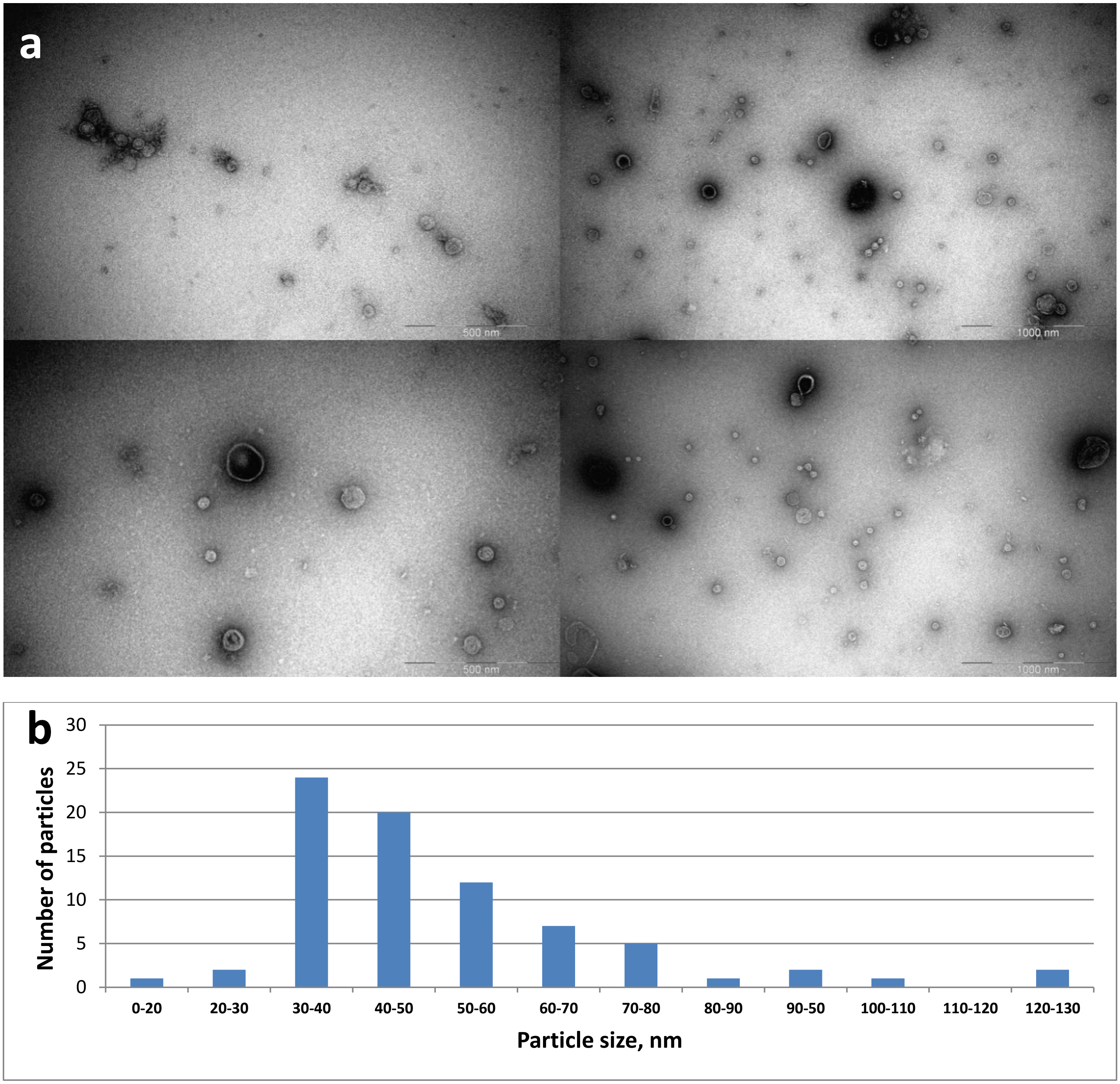

Supplementary Figure 5 TEM analysis of Superose 6 76-78 ml fraction of cmcABC`+D BDPs. a, negative staining TEM micrographs. **b,** particle size distribution, determined with ImageJ software.

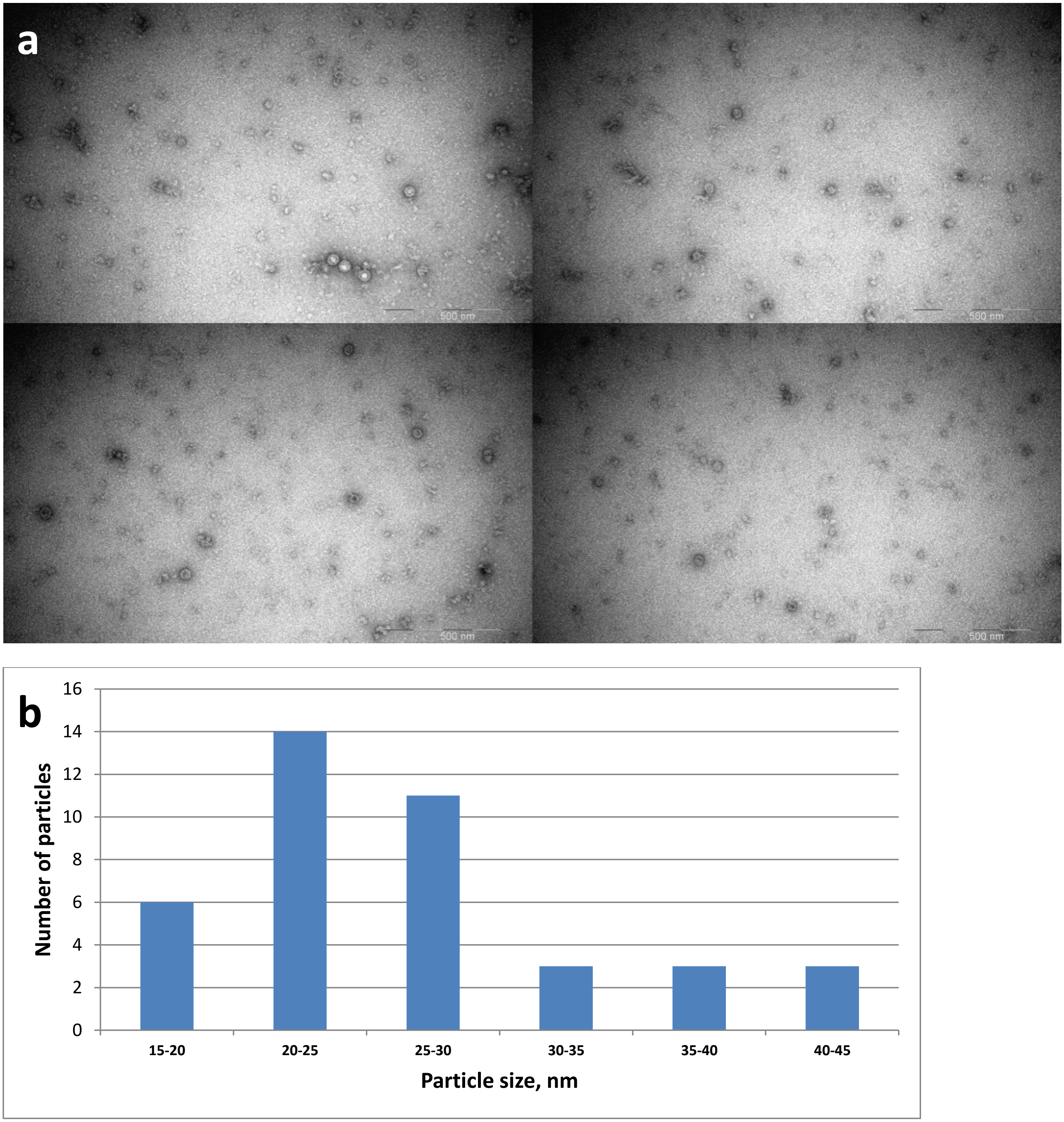

Supplementary Figure 6 TEM analysis of Superose 6 84-86 ml fraction of cmcABC`+D BDPs. a, negative staining TEM micrographs. **b,** particle size distribution, determined with ImageJ software.

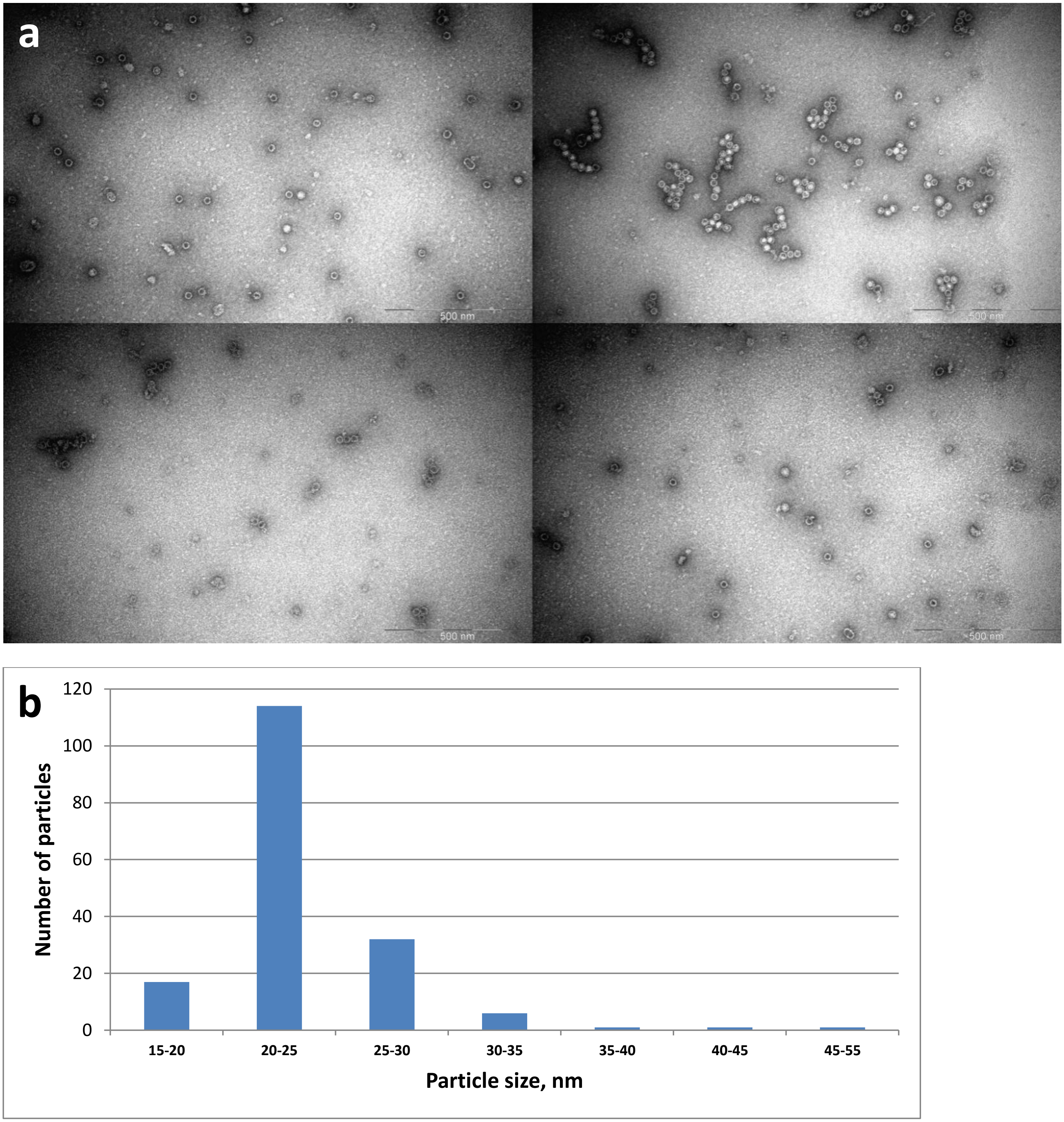

Supplementary Figure 7 TEM analysis of Superose 96-98 ml fraction of cmcABC`+D BDPs. a, negative staining TEM micrographs. **b,** particle size distribution, determined with ImageJ software.

a

```
                                      1         10        20        30        40        50        60        70        80        90        100
     β carboxysome Synechocystis sp.  ....MQLAKVLGTVVSTSKTPNLTGVKLLLVQFLDTKGQ.PLERY...EVAGDVVGAGLNEWVLVARGSAARKERGNGDRPLDAMVVGIIDTVNVASGSLYNKRDDGRLEAAA
                 PDU Citrobacter sp.  ....MHLARVTGVVVSTQKSPSLVGKKLLLVRRVSADGELPASPVSGDEVAVDSVGAGTGELVLLSSGSSARHVFSGPNEAIDLAIVGIVDTLSR
             EUT Salmonella enterica  MEADMKLAVVTGQIVCTVRHQGLAHDKLLMVEMIDAQGN....PDGQCAVAIDSIGAGTGEWVLLVSGSSARQAHRSELSPVDLCVIGIVDEVVAGGKVVFHK
Unknown func. Haliangium ochraceum  ....MVLGKVVGTVVASRKEPRIEGLSLLLVRACDPDGT....PTGGAVVCADAVGAGVGEVVLYASGSSARQTEVTNNRPVDATIMAIVDLVEMGGDVRFRKD
     RMM Mycolicibacterium smegmatis  ....MLRATVTGNVWSTRRIEGIPAGAFLEVEVEGTGSR.........MIAFDVLGSGVGEHVLIAQGSVASSWFTGTPPPIDALIIGSIDTRSDSNPAE
                    GRM1 Clostridium  ....MIAARLIDNVWATRKAESLSGLKFMLAEEIGGIDEGRR......FIVVDIISAGIGDRVIVSTGSSARRMLGDDNIPVDAVVIGIIDEDCNFG
                   GRM5 Ruminococcus  ....MIVGKVVGSIVSTRKSDKLIGQKFMIVEPVHHMKTDLS.....QIVAIDIIGAGIGEYVLVAQGSAARIGCGEETAPIDAAIVGIVDDGAGLE
                     GRM4 Shevanella  ....MQLAKVVGSVVSTQKSASLTGKKLMVVTLVNLDGSTPLFQENTQEVAVDSMGAGEGELVLVSRGSSARKIFPEPNQAIDLAIVGIVDTIDRF
                    GRM3 Clostridium  ....MYMAKVVGNVVATRKDDSLVGYKLMIVKRIDVKENFIGNE....EVAADYVGAGIGDYVLLCKGSAVRVEKQK.....VAIDMAIIGIIDTIDV
          GRM2 Aeromonashydrophila  ....MILAKVTGHVVATQKCDELRGSNLLLITQLDDQQQPMKDRS...WVAVDSVGAGMHDVVLAEEYLALNRDRYK.....AMSVVAIVEKVFRDA
                 GRM2 Serratia sp.  ....MILARVTGHVVATQKSDELRGSNLLLVTALGDDLQAIKDRT...YVAVDSVGAGEDDLVLVEEYFALNKDRYK.....AMSIVAIVEKVHRER
              GRM2 Escherichia coli  ....MILAKVTGHVVATQKCDELRGSNLLLITQLDDNQQLMKDRS...WVAVDSVGAGVHDVVLAEEYFALNKDRYK.....AMSVVAIVEKVFRDA
   GRM2 Klebsiella pneumonia cmcD  ....MILAKVTGHVVATQKCDELRGSNLLLITRLDDKQQPMKDQT...WVAVDNVGAGMHDIVLAEEYFALNKDRYK.....AMSVVAIVEKVFRDTEQE
```

b

```
                                      1         10        20        30        40        50        60        70        80        90        100
     β carboxysome Synechocystis sp. .MSIAVGMIETRGFPAVVEAADSMVKAARVTLVGYEKIGSGRVTVIVRGDVSEVQASVSAGIEAANRVNGGEVLSTHIIARPHENLEYVLPIRYTEEVEQFRTY
                  PDU Citrobacter sp.MQQEALGMVETKGLTAAIEAADAMVKSANVMLVGYEKIGSGLVTVIVRGDVGAVKAATDAGAAAARNVG..EVKAVHVIPRPHTDVEKILPKGIS
             EUT Salmonella enterica ..MEALGMIETRGLVALIEASDAMVKAARVKLVGVKQIGGGLVTAMVRGDVAACKAATDAGAAAAQRIG..ELVSVHVIPRPHGDLEEVFPISFKGDSNI
Unknown func. Haliangium ochraceum .MADALGMIEVRGFVGMVEAADAMVKAAKVELIGYEKTGGGYVTAVVRGDVAAVKAATEAGQRAAERVG..EVVAVHVIPRPHVNVDAALPLGRTPGMDKSA
     RMM Mycolicibacterium smegmatis MSSNAIGLIETKGYVAALAAADAMVKAANVTITDRQQVGDGLVAVIVTGEVGAVKAATEAGAETASQVG..ELVSVHVIPRPHSELGAHFSVSSK
            GRM1_Clostridium sp. MKYDALGMIETKGLVGSIEAADAMVKAANVYLIGKEYVGGGLVTVMVRGDVGAVKAATDAGAAAAQRVG..ELVSVHVIPRPHSEVEVILPSSKEVAK
           GRM5 Ruminococcus sp. MTQEALGMVETRGLTAAIEAADQMCKAANVALVGTEKIGSGLVTVMVRGDVGAVKSAVESGSAAASRLG..ELVATHVIPRPHTDVEKILPVLK
              GRM4 Shevanella sp..MNNALGMIETYGLIGAIVGADAMVKSANVQLVGYEKIGSGLVTVMVRGDVGAVKASVEAGSAAAAGVG..QVKSTHVIPRPHNEVETLLPVYQTATAE
            GRM3 Clostridium sp. MNSDALGMVETKGLVAAIEAADAMVKAANVNLIGYEKIGSGLVTVMVRGDVGAVKAATDAGAASARLVG..EVVSIHVIPRPHMDTEKILPKFV
          GRM2 Aeromonas_hydrophila .MGDALGLIETKGLVACIEAADAMCKSANVELISYENIGSGLVTVMVKGDVGAVKAAVDSGVESAQRIG..EVVTSLVIARPHNDINKIVIKHKA
                 GRM2 Serratia sp. .MGDALGLIETKGLVACVEAADAMCKAANVELIGYENVGSGLVTAMVKGDVGAVKAAVDSGVESAQRVG..EVVTSLVIARPHNDINKIVIKHKA
              GRM2 Escherichia coli .MREALGLIETKGLVACIEAADAMCKAANVELIGYENVGSGLVTAMVKGDVGAVNAAVDSGVEAARRIG..EVVTSRVIARPHNDIEKIASQHKA
    GRM2 Klebsiella pneumonia cmcA .MGDALGLIETKGLVACIEAADAMCKAANVELIGYENVGSGLVTVMVKGDVGAVKAAVDSGVESAQRIG..EVVTSLVIARPHNDINKIVIKHKA
    GRM2 Klebsiella pneumonia cmcB .MGDALGLIETKGLVACIEAADAMCKAANVELIGYENVGSGLVTAMVKGDVGAVKAAVDSGVESAQRIG..EVVTSLVIARPHNDINKIVSHYKIAD
    GRM2 Klebsiella pneumonia cmcC .MKEALGLIETKGLVACIEAADAMCKAANVELIGYENVGSGLVTAMVKGDVGAVNAAVDSGVEAAKRIG..KVVSSRVIARPHNDIEKIAAQHKA
    GRM2 Klebsiellapneumoniae cmcC`.MKEALGLIETKGLVACIEAADAMCKAANVELIGYENVGSGLVTAMVKGDVGAVNAAVDSGVEAAKRIG..KVVSSRVIARPHNDIEKIAGSTKHKSLRPHNA
    GRM2 Klebsiella pneumonia cmcE ..MKSLGVIETRGWVAAIQAVDAACKAAGVTCIGYRKPGSGLVSVCFEGEISAIHTAIERGVAVAGAEH...TVKSLVIARPERCVVEALSNLKGNPPREQKTAEPV
VIPAPEAIVPPAAPNETEDKHPALKKGKKS
```

Supplementary Figure 8 Alignment of GRM2 BMC-H and BMC-P proteins with homologs from other loci. a, alignment of BMC-P protein sequences from β carboxysomes, PDU, Eut, RMM, GRM1, GRM2, GRM3, GRM4, GRM5 and loci of unknown function with *Klebsiella pneumonia* cmcD. The GS-type pore motif is colored in yellow, and the YL and YF pore motifs are marked in cyan. **b,** alignment of PduA-type short BMC-H protein sequences from β carboxysomes, PDU, Eut, RMM, GRM1, GRM2, GRM3, GRM4, GRM5 and loci of unknown function with *Klebsiella pneumoniae* GRM2 cmcA, cmcC` and cmcE. The pore motif is colored yellow, the conservative KRX triad is colored green, and the amino acids involved in a possible salt bridge network on the inner surfaces of the interhexameric contacts are colored cyan. Data were obtained from Axen et al.[1]

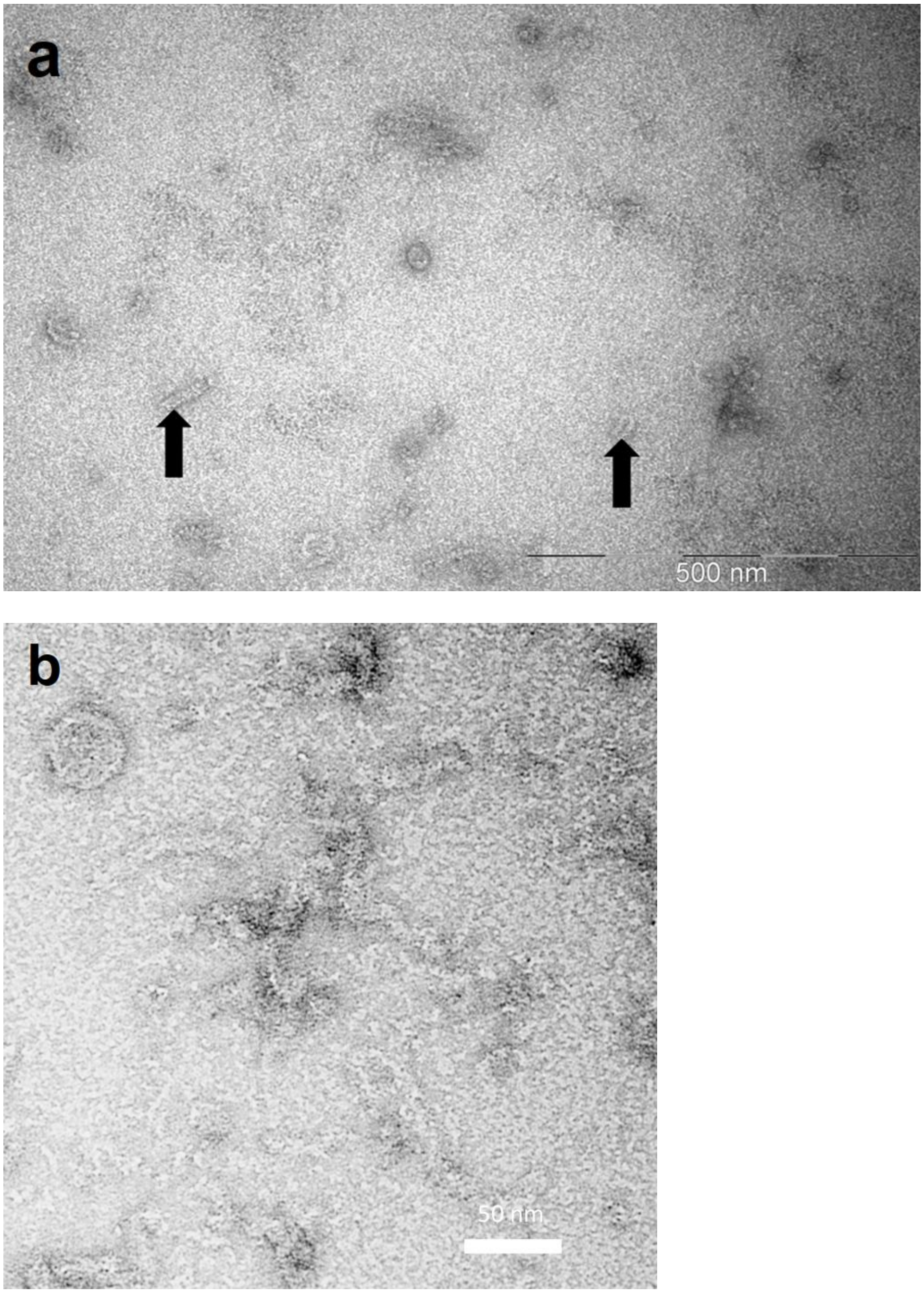

**Supplementary Figure 9 TEM analysis of a, cmcABC` and b, cmcE+D BDPs.** Both tubular and round particles can be observed. Fractions containing 80-84 ml from cmcABC` and cmcE+D 94-96 ml Superose 6 gel filtration were analyzed.

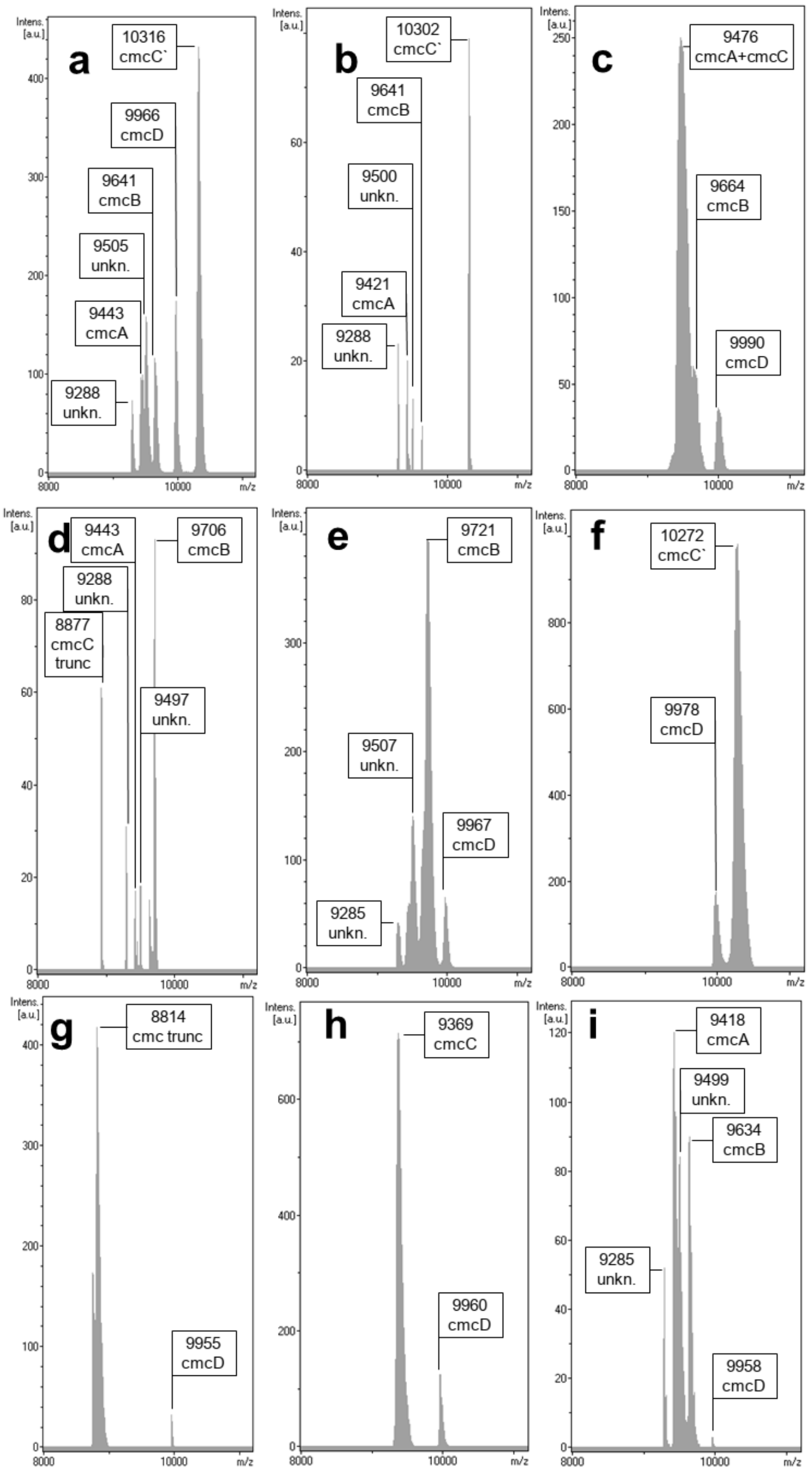

Expected m/z values

cmcA	9415
cmcB	9624
cmcC	9414
cmcC`	10293
$cmcC_{trunc}$	8879
cmcD	9949

Supplementary Figure 10 MALDI-TOF analyses of BDPs. a, cmcABC`+cmcD small particle zone (94-96 ml fraction of Superose 6 gel filtration chromatography).**b,** cmcABC`+cmcD large particle zone (58-60 ml fraction of Superose 6 gel filtration chromatography). **c,** cmcABC+cmcD small particle zone (94-96 ml fraction of Superose 6 gel filtration chromatography). **d,** $cmcABC_{trunc}$+cmcD large particle zone (58-60 ml fraction of Superose 6 gel filtration chromatography). **e,** $cmcABC_{trunc}$+cmcD small particle zone (94-96 ml fraction of Superose 6 gel filtration chromatography).**f,** cmcC`+cmcD small particle zone (94-96 ml fraction of Superose 6 gel filtration chromatography). **g,** $cmcC_{trunc}$+cmcD small particle zone (94-96 ml fraction of Superose 6 gel filtration chromatography). **h,** cmcC+cmcD large particle zone (58-60 ml fraction of Superose 6 gel filtration chromatography). **i,** cmcAB+cmcD small particle zone (94-96 ml fraction of Superose 6 gel filtration chromatography).

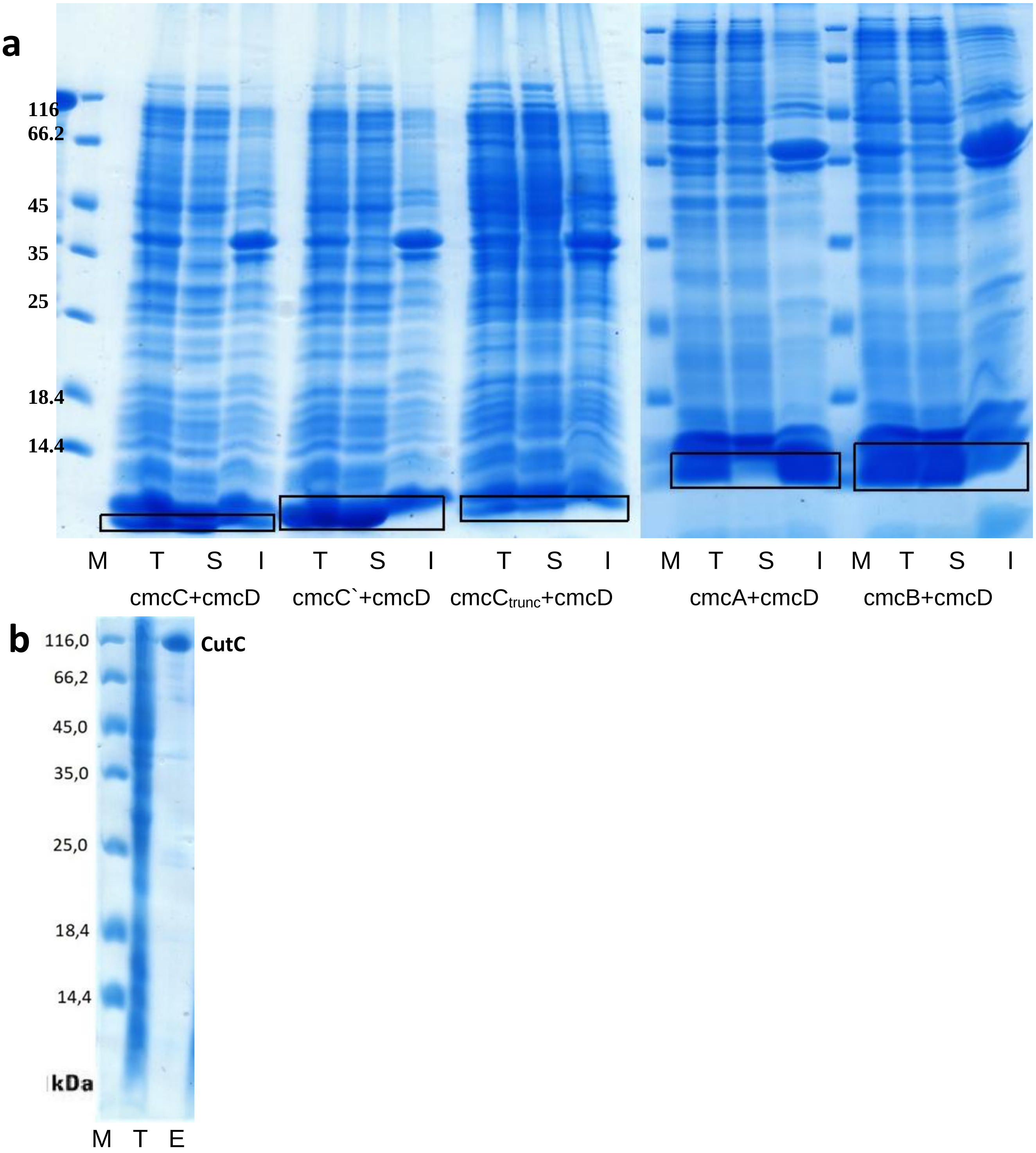

Supplementary Figure 11 Expression level and solubility SDS-PAGE analysis of cmcC+cmcD, cmcC`+cmcD, cmcC$_{trunc}$+cmcD, cmcA+cmcD and cmcB+cmcD constructs and SDS-PAGE analysis of pull-down assay of H6-CutC+CutO co-expression. a,** cmcC, cmcC`, cmcC$_{trunc}$, cmcA and cmcB zones are framed, cmcC` is very close to cmcD and both are framed. Total lysate (T), supernatant after centrifugation at 16000 g (S) and insoluble fraction in pellet (I). M – marker, sizes are measured in kDa. **b, SDS-PAGE analysis of H6x-CutC+CutO co-expression His-trap Ni^{2+} affinity chromatography elution fraction (E) and total lysate (T). Only CutC band is present in elution fraction, no CutO is observable. M – marker, sizes are measured in kDa.

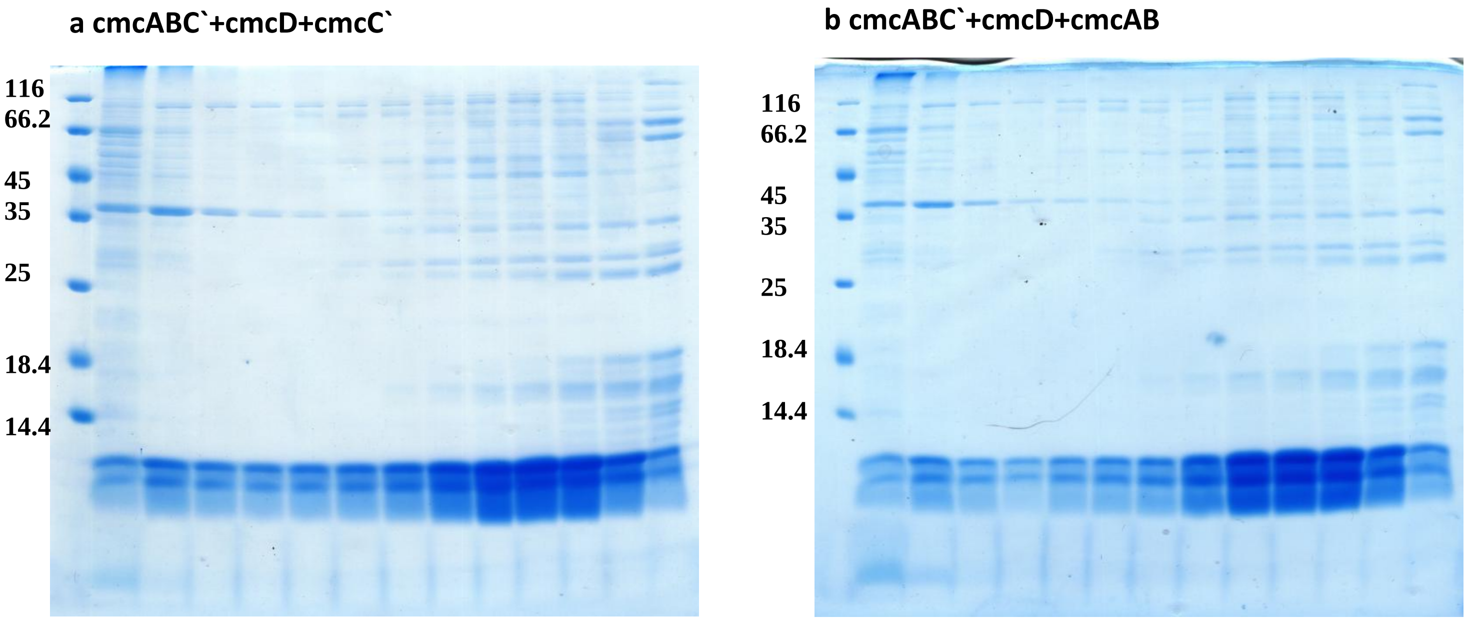

Supplementary Figure 12 SDS-PAGE analysis of Superose 6 gel filtration chromatography processes. a, cmcABC`+cmcD+cmcC 60-108 ml. **b**, cmcABC`+cmcD+cmcAB 60-108 ml.

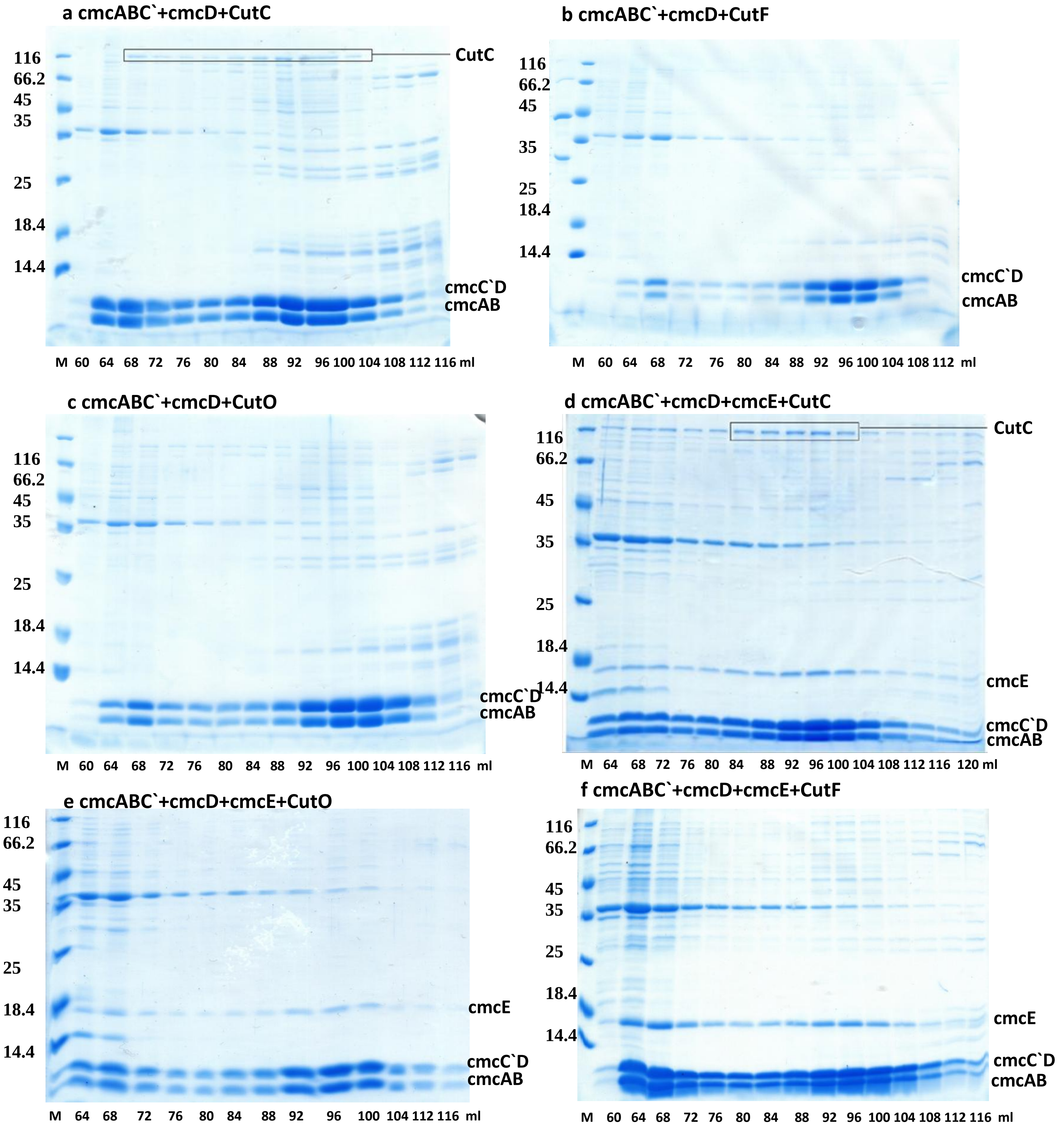

Supplementary Figure 13 SDS-PAGE analysis of Superose 6 gel filtration chromatography processes, corresponding protein bands are identified. a, cmcABC`+cmcD +CutC 60-116 ml. **b**, cmcABC`+cmcD +CutF 60-112 ml. **c,** cmcABC`+cmcD +CutO 60-112 ml. **d,** cmcABC+cmcD+cmcE 64-120 ml, **e,** cmcABC`+cmcD+cmcE+CutO 64-116 ml. **f,** cmcABC`+cmcD+cmcE+CutF 60-116 ml. M – marker, sizes are measured in kDa.

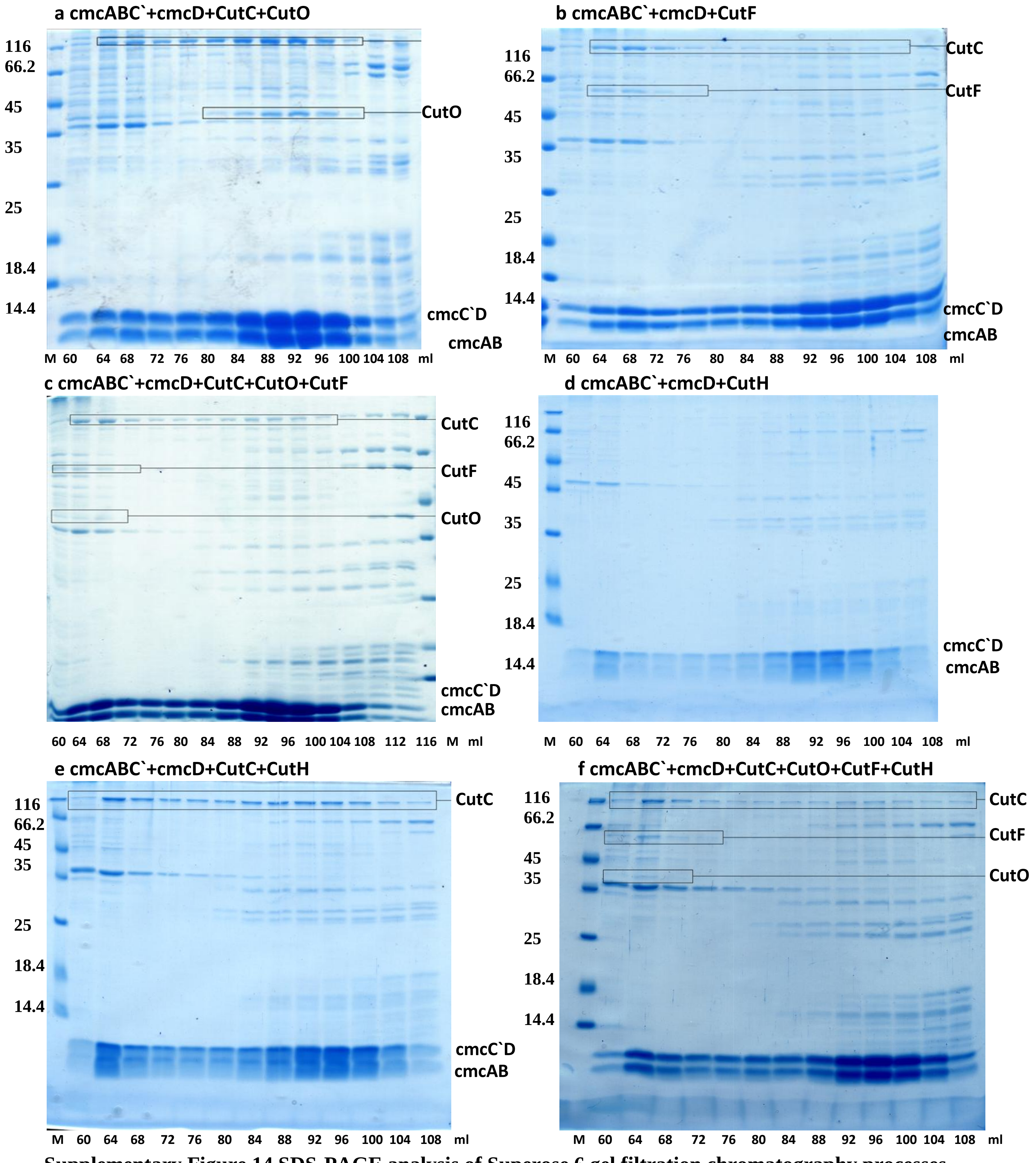

Supplementary Figure 14 SDS-PAGE analysis of Superose 6 gel filtration chromatography processes, corresponding protein bands are identified. a, cmcABC`+cmcD+CutC+CutO 60-108 ml. **b**, cmcABC`+cmcD +CutC+CutF 60-108 ml. **c,** cmcABC`+cmcD+CutC+CutO+CutF 60-116 ml. **d,** cmcABC`+cmcD+CutH 60-108 ml. **e,** cmcABC`+cmcD+CutC+CutH 60-108 ml. **f,** cmcABC`+cmcD+CutC+CutO+CutF+CutH 60-108 ml. M – marker, sizes are measured in kDa.

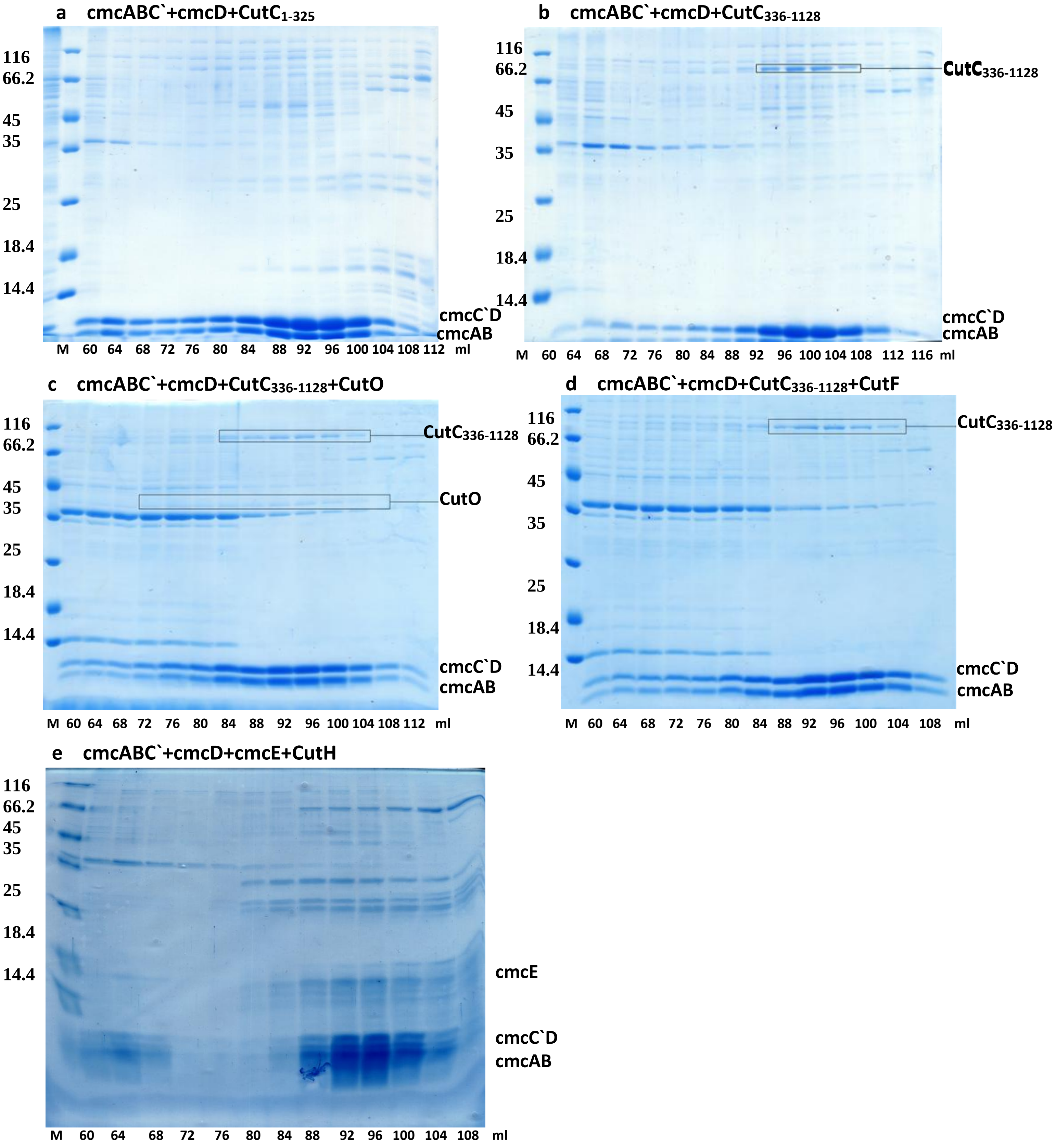

Supplementary Figure 15 SDS-PAGE analysis of Superose 6 gel filtration chromatography processes, corresponding protein bands are identified. a, cmcABC`+cmcD+CutC$_{1-325}$ 60-112 ml. **b,** cmcABC`+cmcD+CutC$_{336-1128}$ 60-116 ml. **c,** cmcABC`+cmcD+CutC$_{336-1128}$+CutO 60-122 ml. **d,** cmcABC`+cmcD+CutC$_{336-1128}$+CutF 60-108 ml. **e,** cmcABC`+cmcD+cmcE+CutH 60-108 ml. M – marker, sizes are measured in kDa.

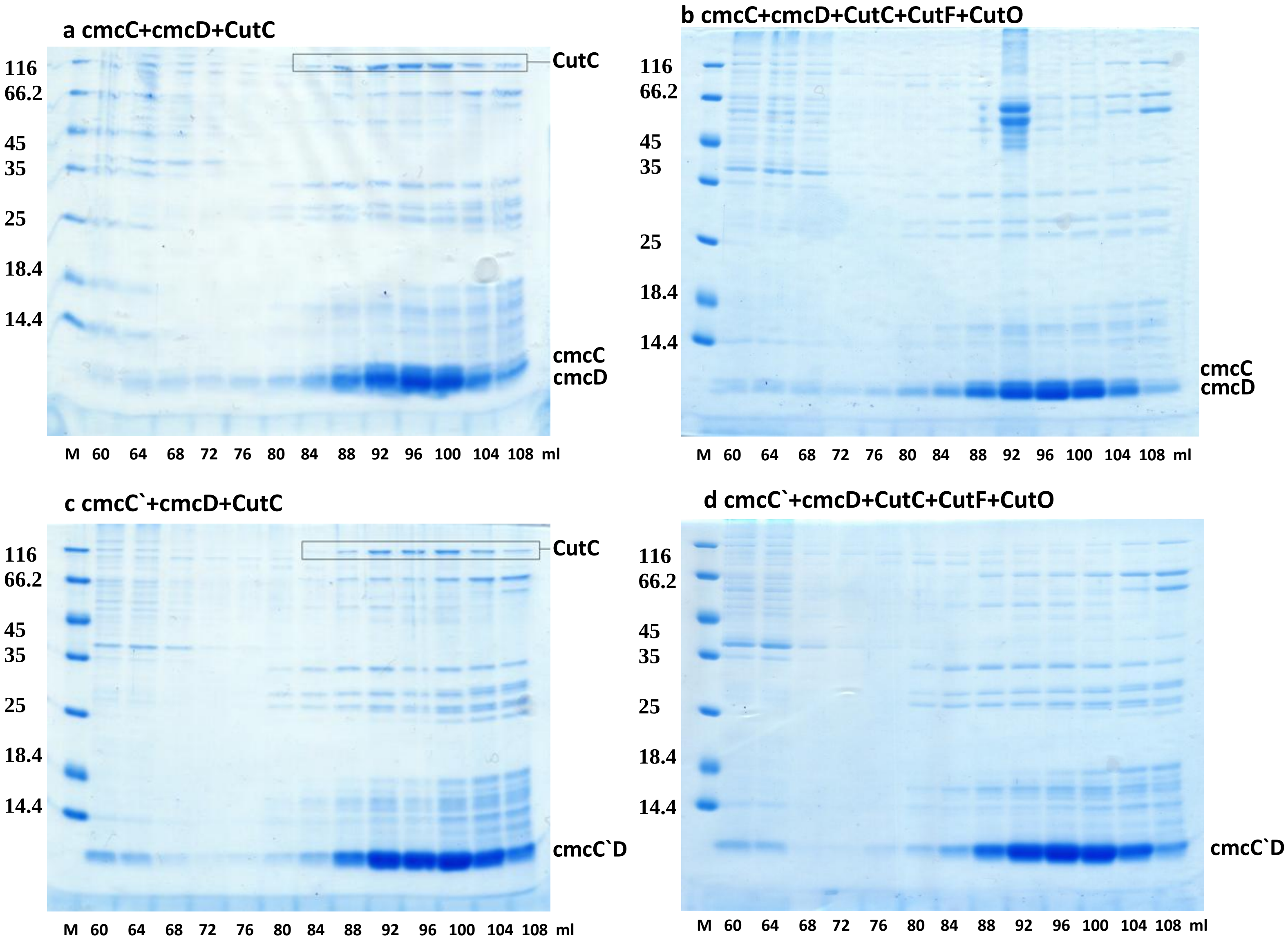

Supplementary Figure 16 SDS-PAGE analysis of Superose 6 gel filtration chromatography processes. a, cmcC+cmcD+CutC 60-108 ml. **b**, cmcC+cmcD+CutC+CutF+CutO 60-108 ml. **c,** cmcC`+cmcD+CutC 60-108 ml. **d**, cmcC`+cmcD+CutC+CutF+CutO 60-108 ml. M – marker, sizes are measured in kDa.

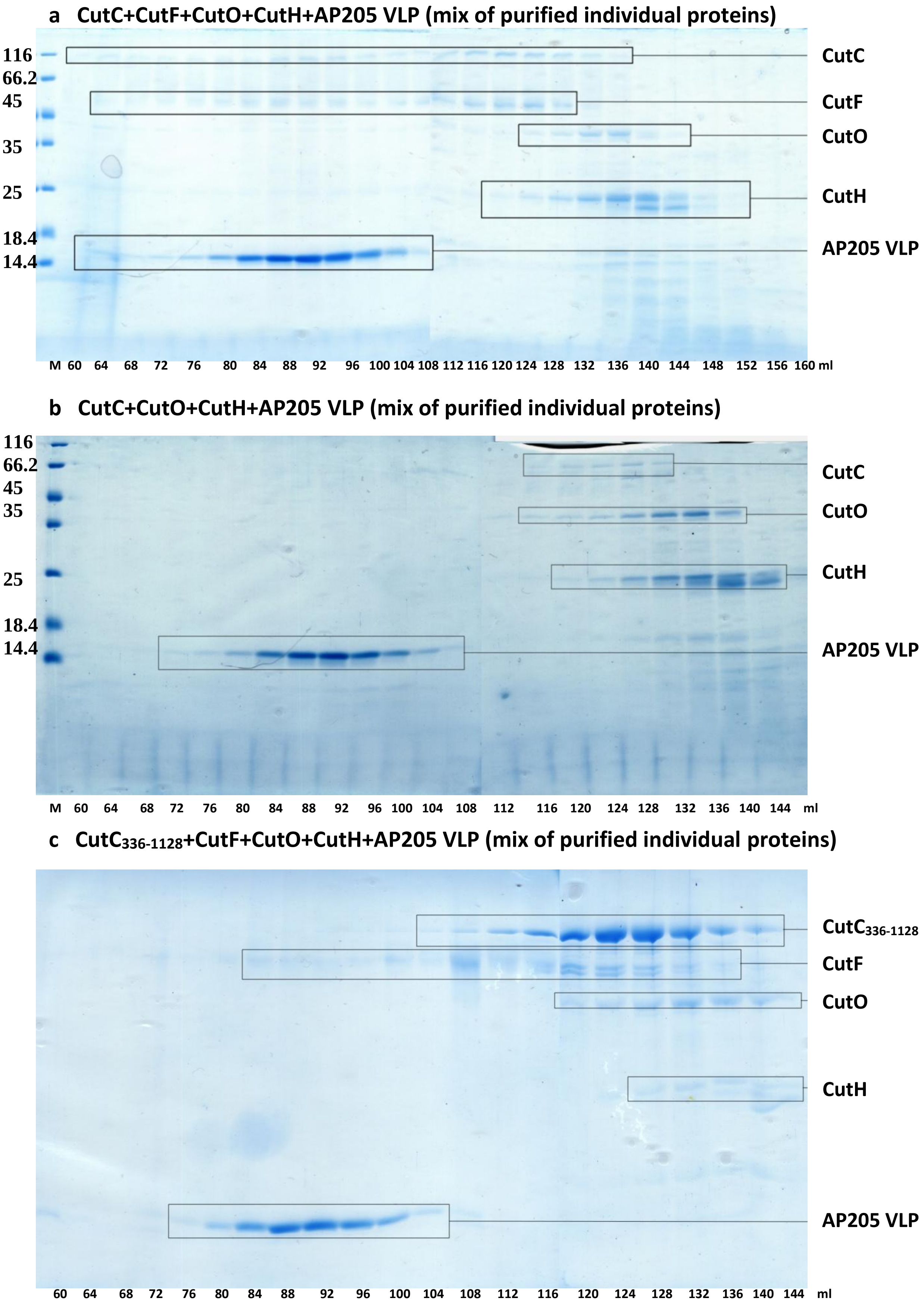

Supplementary Figure 17 SDS-PAGE analysis of Superose 6 gel filtration chromatography processes. a, mixture of H6x tagged, purified CutC, CutF, CutO, CutH and AP205 VLP, 60-160 ml. **b,** mixture of H6x tagged, purified CutC, CutO, CutH and AP205 VLP, 60-160 ml. **c,** mixture of H6x tagged, purified $CutC_{336\text{-}1128}$, CutF, CutO, CutH and AP205 VLP, 60-160 ml. M – marker, sizes are measured in kDa.

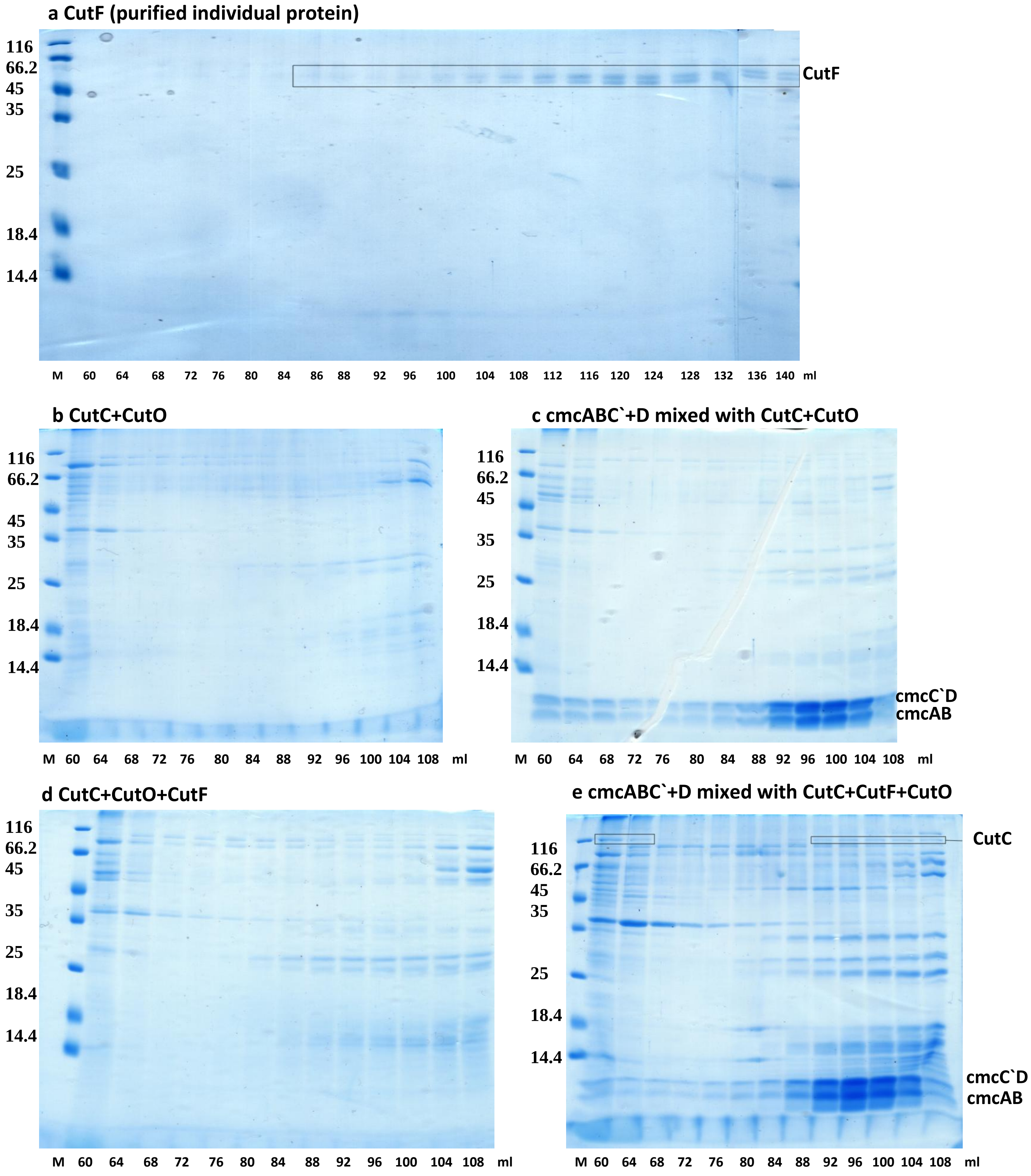

Supplementary Figure 18 SDS-PAGE analysis of Superose 6 gel filtration chromatography processes. a, purified H6x tagged CutH, 60-140 ml. **b,**CutC, 60-108 ml. **c,**CutC biomass mixed with cmcABC`+D biomass. **d,**CutC+CutO+CutF, 60-108 ml. **e,**CutC+CutO+CutF biomass mixed with cmcABC`+D biomass.

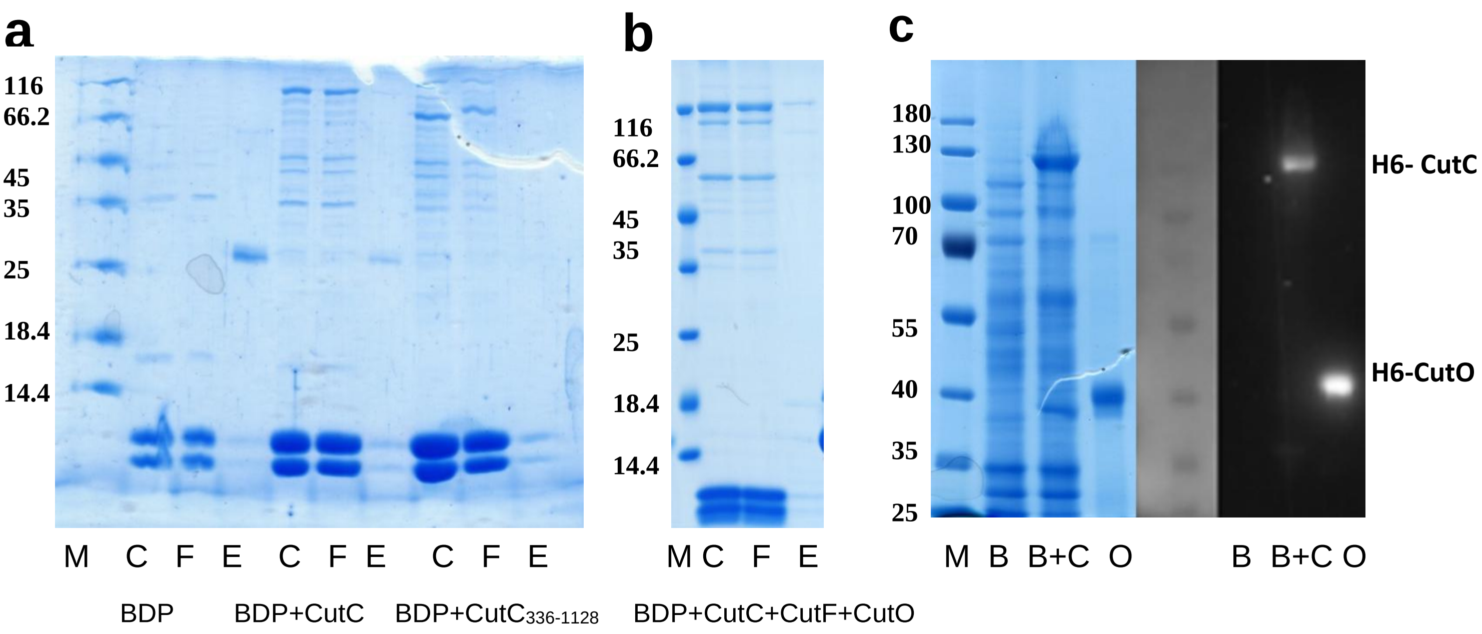

Supplementary Figure 19 SDS-PAGE and Western blot analysis of BDP His-trap Ni^{2+} affinity chromatography fractions. a, control (C), flow-through (F) and elution (E) fractions obtained from analytic chromatography of small type BDPs. BDPs without encapsulated cargo (BDP), BDPs with encapsulated H6x-CutC (cmcABC+D +CutC) and BDPs with encapsulated H6-$CutC_{336-1128}$ (cmcABC+D+$CutC_{336-1128}$). **b,** control (C), flow-through (F) and elution (E) fractions obtained from analytic chromatography of large type BDPs particles with encapsulated H6x-CutC+CutF+CutO (cmcABC+D+CutC+CutF+CutO). The analyzed protein was in the large particle Superose 6 gel filtration peak. M – marker, sizes are measured in kDa. **c,** SDS-PAGE (left) and anti-His6x tag Western blot (right) analysis of small type BDPs (B), small type BDPs with encapsulated CutC (B+C) and control his6x-tagged CutO (O). M – marker, sizes are measured in kDa.

a

MIELDNDLQSRQNARELVRNAKKAQAMLATFSQQQIDAIVK**NVAQEAAHHAEALAK**MAAEETGFGNWQDK
VLKNRFASLR**VYDAIK**DMKTVGIIHDDPVKKVMDVGVPLGVICALVPSTNPTSTVIYKALIALKAGNAII
FSPHPGARQCSWKAIEIVKRAAEAAGAPEGCVDGITQLTLEATSELMHSKDVSLILATGGEGMVRAAYAS
GTPTISGGPGNGPAFIER**SADIHHAVK**DIITSKTFDNGVICASEQSIIVEGCIYDEVHRELEAQGAYFMN
EDEAAKMAALLLRPNGTINPKVVGKTALYLSQMAGFCVPASTKVLIAEQTTVSPK**NPYSR**EKLCPVLGLY
VAEDWKAACHRVVELLTNEGLGHTLVIHTRNQDVIRQFSLEKPVNRILINTPAALGGIGATTNISPALTL
GCGAVGGGSSSDNVGPMNLLNIRKVGYGVRSIDELRAPGSRPEPQPTIVSPASDPQRSILDDVRFNAPAN
AAPAR**SAGSDDR**FASAGAASMEGEINEQNVERVIRQVLERLAK

M/z observed	M/z expected	Peptide
1559.8953	1559.7924	NVAQEAAHHAEALAK
976.2629	977.5163	SADIHHAVK
708.2392	708.3926	VYDAIK
707.1857	707.2954	SAGSDDR
636.2541	636.31	NPYSR

b

MSEFLLKPRICFGQDALSVLNELSAR**SVLLVTDQAMVKFGLAERVTALLR**QR**GIAWQMWDDVVADPDIAT**
VVRGMKLMDNHYPDLVIALGGGSVIDAAKAVIFSLAQTRPQANRPRPCFVAIPTTSGTGSEVTAFSVVKA
NAEKLVLVDASLLPDIAILDPALVTSVPPAITADTGMDVLCHALEAYVSRAASDFSDALAEK**VVQQVFRY**
LPTCWRSGDNLLAREKMHNASCMAGMAFTNASLGITHSLAHALGGVFRVPHGRANALLMAHVVAWNADVD
GQCDTLAAHKYAR**LAHLLDLPAASPR**QGVASLLVAIQALKEEMNMPSGISDTGIDAPEFDRRLPEMVGQA
LR**DSCTPTNPR**APDANALTELYRQAWHGQQTSPGGAPLARAYG

M/z observed	M/z expected	Peptide
2358.8623	2357.1594	GIAWQMWDDVVADPDIAT
1373.5954	1373.7899	LAHLLDLPAASPR
1303.5294	1303.7290	SVLLVTDQAMVK
990.6034	990.4309	DSCTPTNPR
939.7214	938.4553	YLPTCWR
877.1389	875.5097	VVQQVFR
695.1060	692.3726	FGLAER
675.3118	672.4403	VTALLR

Supplementary Figure 20 MALDI-TOF peptide mass fingerprinting analysis of CutF and CutO zones a, CutF (Supplementary Figure 5c). **b,** CutO (Supplementary Figure 5a). Identified peptides are colored in red.

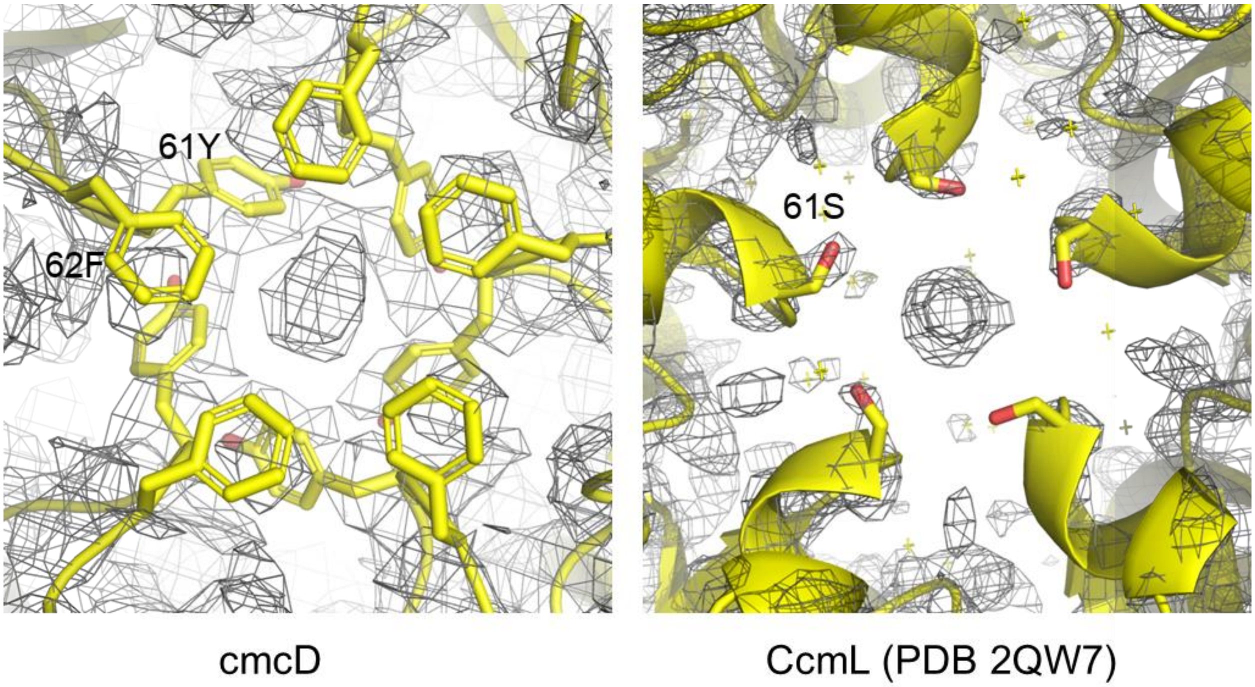

Supplementary Figure 21 Comparison of cmcD and CcmL (PDB 2QW7) pore structures. Map for cmcD rendered at sigma 3 level, map for CcmL rendered at sigma 2 level.

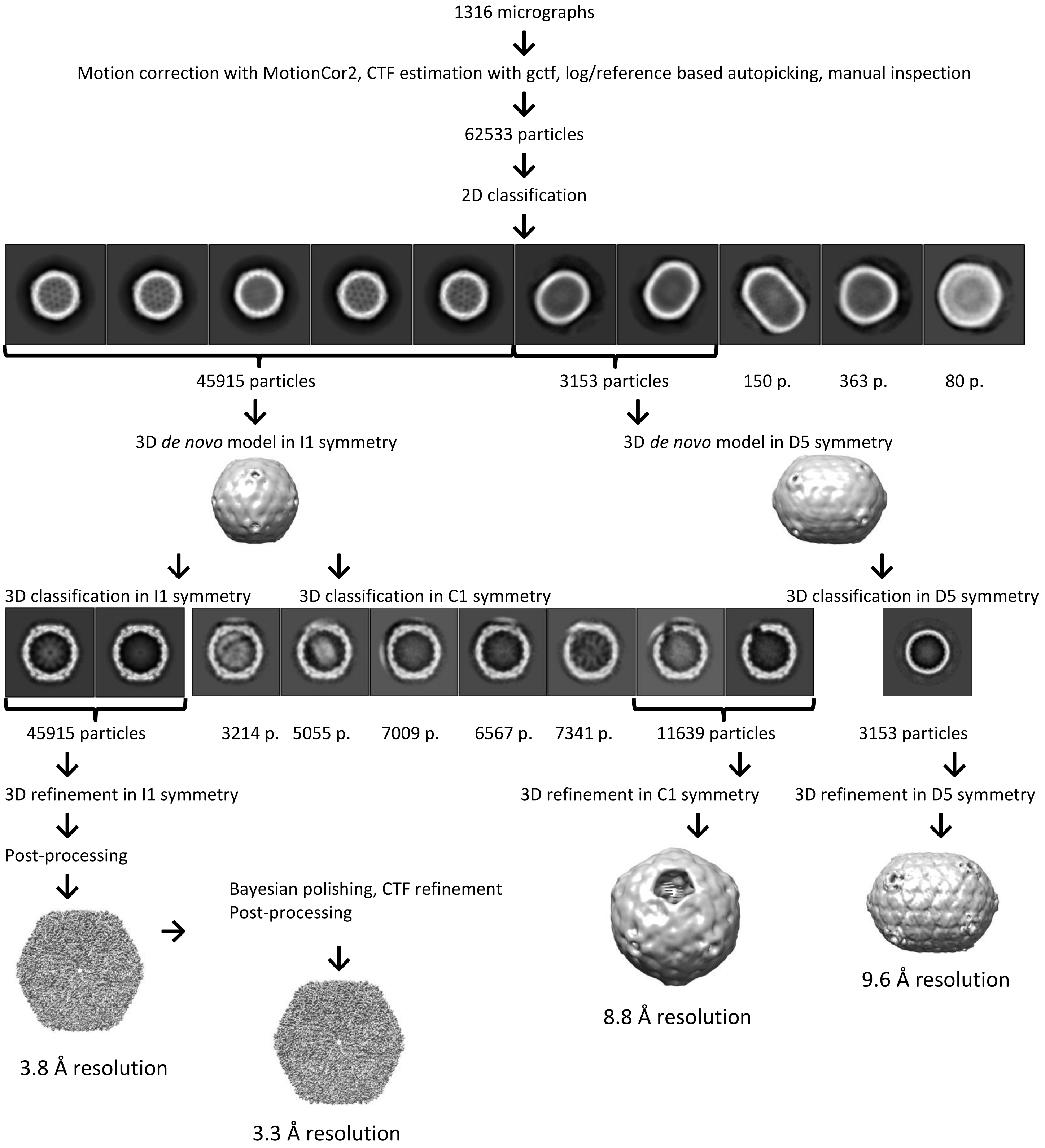

Supplementary Figure 22 Cryo-EM analysis of cmcABC`+cmcD+CutC BMC particles from Superose 6 gel filtration small particle peaks. Image processing workflow is described in the Materials and Methods section.

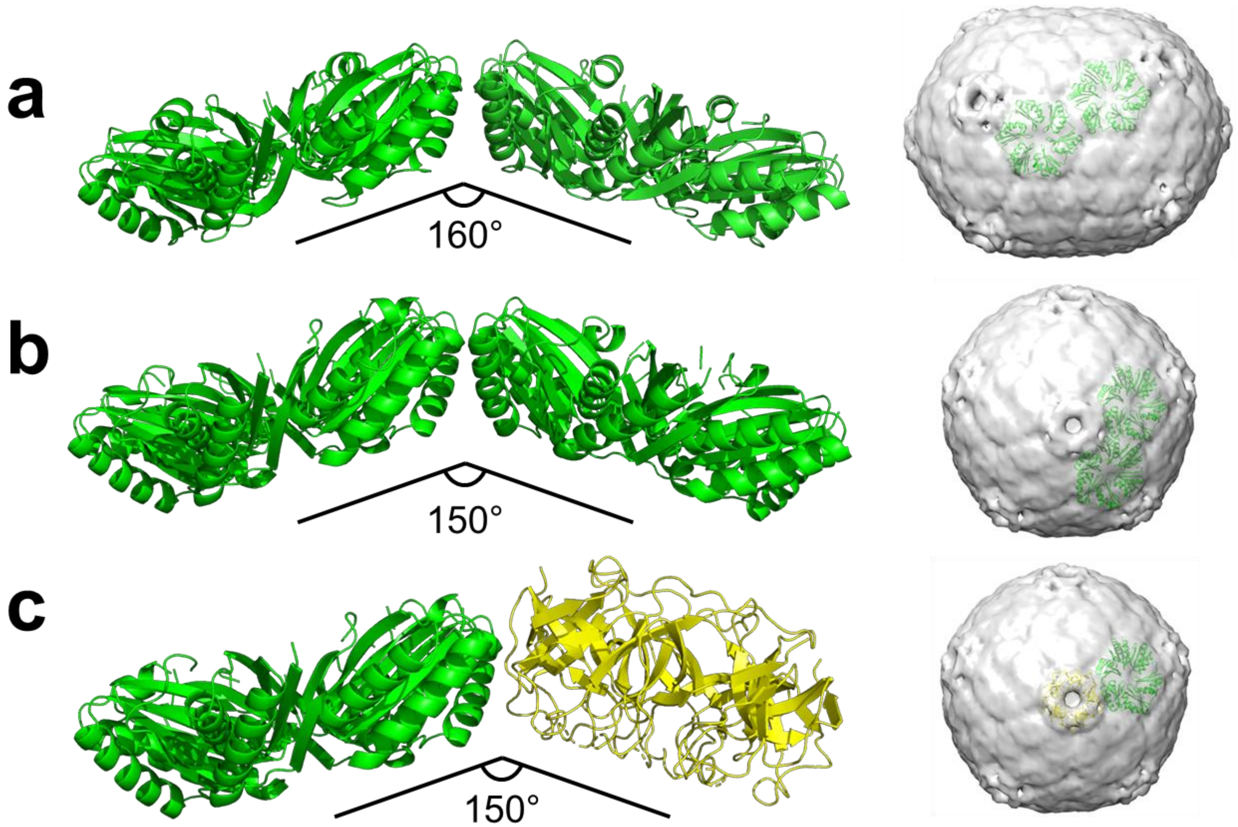

Supplementary Figure 23 Hexamer-hexamer and pentamer-hexamer contacts formed in GRM2 BMC particles. cmcC` hexamers are colored green, and cmcDpentamers are colored yellow. **a,** pT=4,Q=7 hexamer-hexamer contacts. Two hexameric cmcC` atomic models were fitted in a low-resolution pT=4,Q=7 map in UCSF Chimera. **b,** pT=4 hexamer-hexamer contacts. **c,** pT=4 pentamer-hexamer contacts.

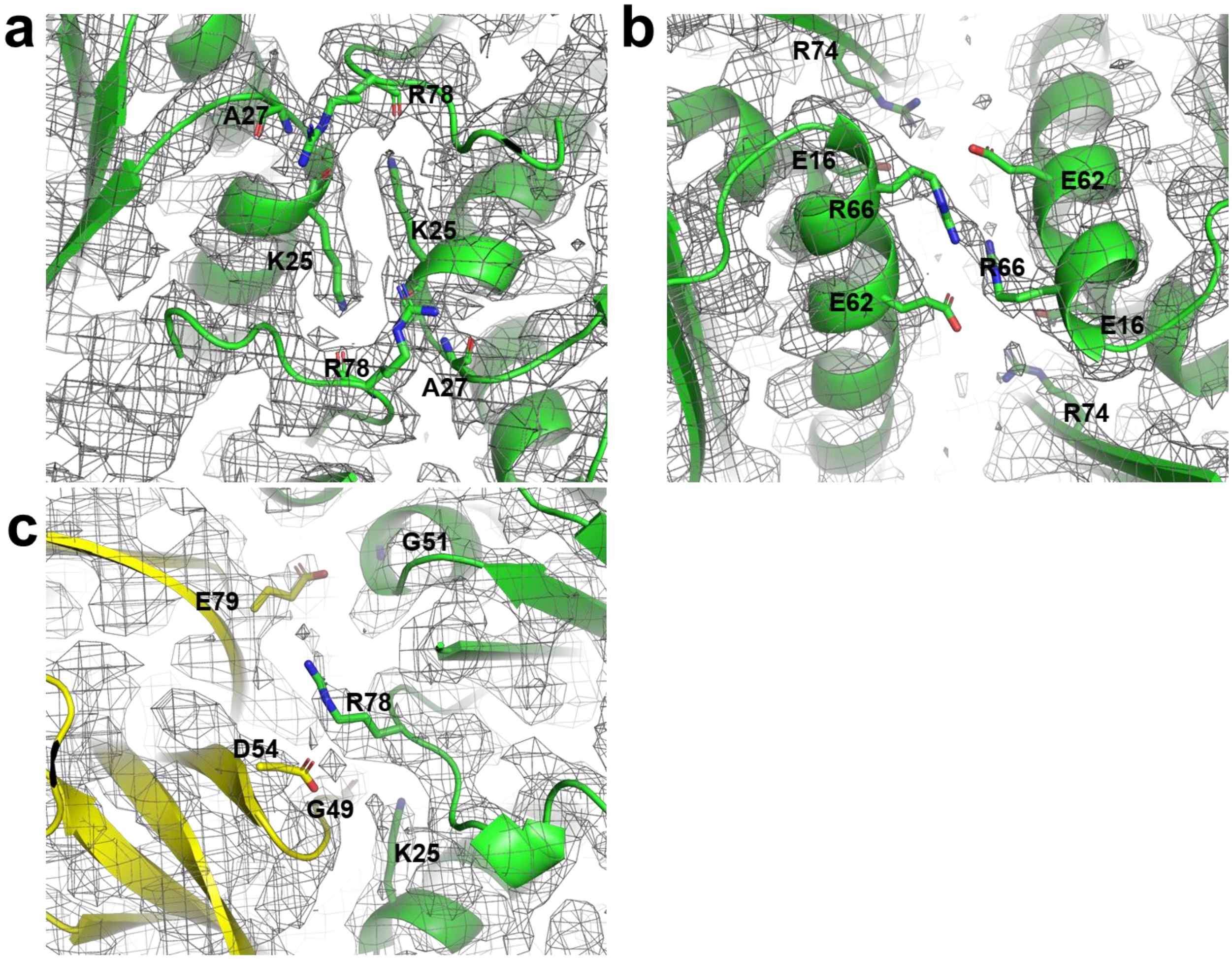

Supplementary Figure 24 Detailed view of hexameric-hexameric (a and b) and pentameric-hexameric interfaces (c) with displayed electron density. Maps are displayed at the sigma-3 level.

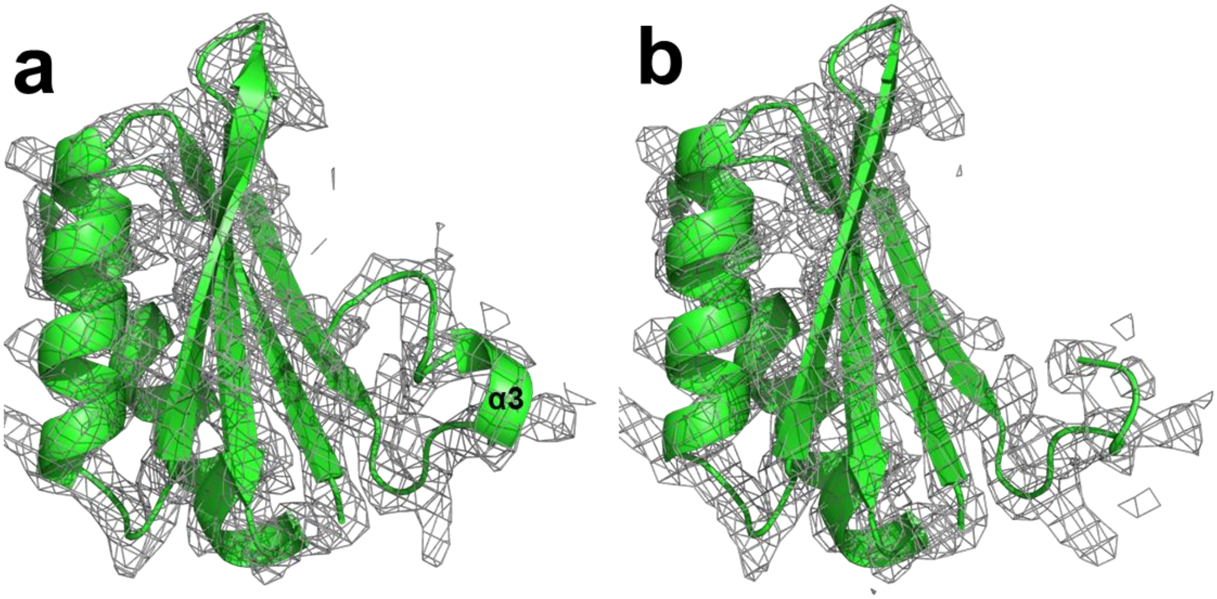

Supplementary Figure 25 Comparison of cmcC` monomer structures involved in different contacts. a, hexamer-pentamercontact. **b,** hexamer-hexamer contact. Cryo-EM maps are displayed at the sigma-2 level.

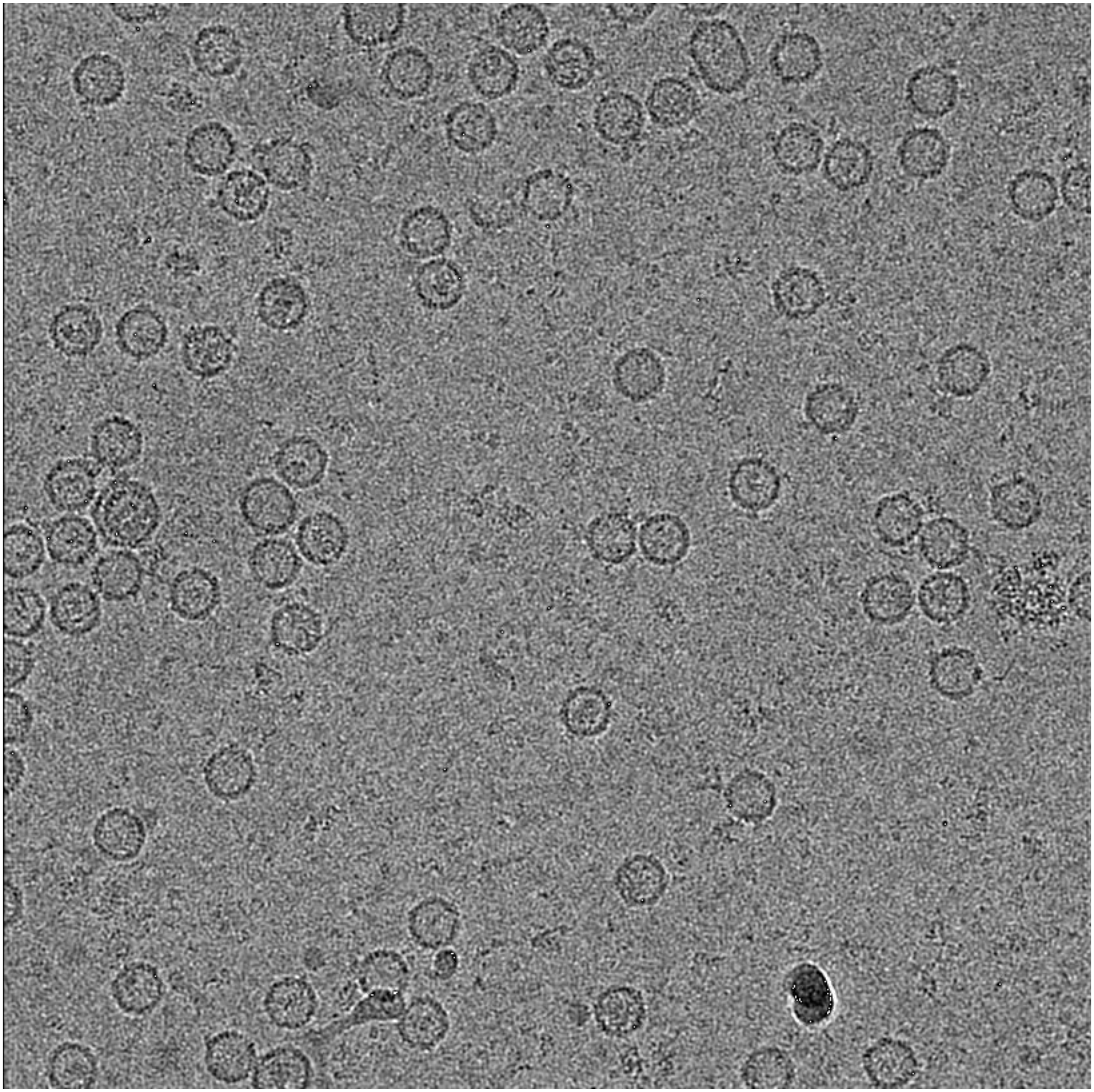

Supplementary Figure 26 An exemplatory cryo-EM micrograph of BDPs.

Supplementary Table 1 Primer sequences.

CutC (PagI/NcoI+HindIII), T7-1	Fw ATATTCATGACGGCACACTACAACTTAACGCCGC
	Rv AATTAAGCTTTTAGAACTTCTCAATCACCGTACGGC
CutF (NcoI+HindIII), T7-1	Fw ATATCCATGGTTGAACTGGATAACGATTTGCAGTCC
	Rv ATATAAGCTTTTACTTAGCAAGGCGCTCCAGC
CutF (NdeI+XhoI), T7-2	Fw ATATCATATGATTGAACTGGATAACGATTTGCAGTCC
	Rv ATATCTCGAGTTACTTAGCAAGGCGCTCCAGC
CutO (BglII/BamHI+HindIII), T7-1	Fw ATATAGATCTATGAGTGAATTTTTACTGAAACCGCG
	Rv ATATAAGCTTTTACCCGTAGGCCCGCGC
CutO (NdeI+XhoI), T7-2	Fw ATATCATATGAGTGAATTTTTACTGAAACCGCG
	Rv ATATCTCGAGTTACCCGTAGGCCCGCGC
CutH (NcoI+HindIII), T7-1	FwATATCCATGGTCGACACCCTGGTTCGCG
	RvATATAAGCTTTTAGCCGATGAGCGTCACCTG
CutC 336-1128 (PagI/NcoI+HindIII), T7-1	Fw ATATTCATGAGCGGCTTAACCCCGCGTATGC
	Rv AATTAAGCTTTTAGAACTTCTCAATCACCGTACGGC
CutC 1-226 (PagI/NcoI+HindIII), T7-1	Fw ATATTCATGACGGCACACTACAACTTAACGCCGC
	Rv AATTAAGCTTTTAGTGGCTGACCGGCTGTACGC
cmcABC (NcoI+HindIII), T7-1	Fw ATATCCATGGGTGATGCATTGGGGCTTATCGAAACCAAAG
	Rv ATATAAGCTTTTATGCTTTGTGCTGCGCCGCGATTTTTTCG
cmcABC\` (NcoI+HindIII), T7-1	Fw ATATCCATGGGTGATGCATTGGGGCTTATCGAAACCAAAG
	Fw ATATAAGCTTTTATGCTTTGTGCTGCCCGCGATTTTTTCG
cmcD (NdeI+XhoI), T7-2	Fw ATATCCATGGTGCTCGCAAAGGTAACCGGCC
	RV ATATAAGCTTTTACTCCTGTTCGGTGTCCCGAAAC
cmcE (NcoI+HindIII), T7-1	Fw ATATCCATGGCCAAAAGTTTAGGCGTAATTGAAACGC
	Rv ATATAAGCTTTTATGACTTCTTCCCTTTCTTCAGCGCCGG
cmcA (NcoI+HindIII), T7-1	Fw ATATCCATGGGTGATGCATTGGGGC
	Rv ATATAAGCTTTTAGGCCTTGTGTTTAATGACGATTTTG
cmcC (NcoI+HindIII), T7-1	Fw ATATCCATGGCCAAAGAAGCGCTTGGTCTTATCGAAAC
	Rv ATATAAGCTTTTATGCTTTGTGCTGCGCCGCGATTTTTTCG
cmcC\` (NcoI+HindIII), T7-1	Fw ATATCCATGGCCAAAGAAGCGCTTGGTCTTATCGAAAC
	Rv ATATAAGCTTTTAAGCATTATGCGGCCGCAAACTTTTATG
cmcCtrunc (NcoI+HindIII), T7-1	Fw ATATCCATGGCCAAAGAAGCGCTTGGTCTTATCGAAAC
	Rv ATATAAGCTTTTAGATTTTTTCGATGTCGTTATGTGGAC
whole prsf-CutO-Duet1 region for insertion in prsf-CutC-CutFXhoI site	Fw TATACTCGAGGATCGATCTCGATCCCGCG
	Rv ATATCTCGAGTTACCCGTAGGCCCGCGC

GenBank™ accession number ARRZ01000032.1 entry[2] was used for primer design.

Supplementary Table 2 Cryo-EM data collection, refinement and validation statistics.

	pT=4 BMC derived particles	pT=4 BMC derived particles with one missing penameric unit	pT=4,Q=7 BMC particles
	EMDB EMD-4595	EMDB EMD-4597	EMDB EMD-4596
	PDB 6QN1		
**Data collection and processing**			
Magnification	120000	120000	120000
Voltage (kV)	200	200	200
Electron exposure ($e^{-}/Å^2$)	60	60	60
Defocus range (μm)	-1.4 to -3	-1.4 to -3	-1.4 to -3
Pixel size (Å)	1.23	1.23	1.23
Symmetry imposed	I	C1	D5
Initial particle images (no.)	62533	62533	62533
Final particle images (no.)	45915	11639	3153
Map resolution (Å)	3.3	8.75	9.64
FSC threshold	0.143	0.143	0.143
Map resolution range (Å)	3.3-35	8.75-35	9.64-35

**Refinement**			
Initial model used (PDB code)	4QIV and 4N8X		
Model resolution (Å)	3.3		
FSC threshold	0.143		
Map sharpening *B* factor ($Å^2$)	-150		
Model composition			
Non-hydrogen atoms	141480		
Protein residues	19740		
Ligands			
B factors ($Å^2$)			
Protein	30		
Ligand			
R.m.s. deviations			
Bond lengths (Å)	0.007		
Bond angles (°)	0.62		
Validation			
MolProbity score	1.56		
Clashscore	4.44		
Poor rotamers (%)	0.4		
Ramachandran plot			
Favored (%)	95.1		
Allowed (%)	4.9		
Disallowed (%)	0		

Supplementary references

1. Axen, S. D., Erbilgin, O., Kerfeld, C. A. A taxonomy of bacterial microcompartment loci constructed by a novel scoring method. PLOS Comput. Biol. 10, e1003898 (2014).
2. Wright M.S., Perez F., Brinkac L., Jacobs M.R., Kaye K., Cober E., van Duin D., Marshall S.H., Hujer A.M., Rudin S.D., Hujer K.M., Bonomo R.A., Adams M.D. Population structure of KPC-producing Klebsiella pneumoniae isolates from midwestern U.S. hospitals. Antimicrob Agents Chemother 58, 4961-5 (2014).
